# Wastewater surveillance of antibiotic-resistant bacterial pathogens: A systematic review

**DOI:** 10.3389/fmicb.2022.977106

**Published:** 2022-12-15

**Authors:** Ananda Tiwari, Paula Kurittu, Ahmad I. Al-Mustapha, Viivi Heljanko, Venla Johansson, Ocean Thakali, Shyam Kumar Mishra, Kirsi-Maarit Lehto, Anssi Lipponen, Sami Oikarinen, Tarja Pitkänen, WastPan Study Group, Annika Länsivaara, Annamari Heikinheimo

**Affiliations:** Faculty of Medicine and Health Technology, Tampere University, Tampere, Finland; Expert Microbiology Unit, Finnish Institute for Health and Welfare, Kuopio, Finland; Expert Microbiology Unit, Finnish Institute for Health and Welfare, Helsinki, Finland; Infectious Disease Control and Vaccinations Unit, Finnish Institute for Health and Welfare, Helsinki, Finland; ^1^Department of Food Hygiene and Environmental Health, Faculty of Veterinary Medicine, University of Helsinki, Helsinki, Finland; ^2^Department of Veterinary Public Health and Preventive Medicine, Faculty of Veterinary Medicine, University of Ibadan, Ibadan, Nigeria; ^3^Department of Veterinary Services, Kwara State Ministry of Agriculture and Rural Development, Ilorin, Nigeria; ^4^Department of Civil Engineering, University of Ottawa, Ottawa, ON, Canada; ^5^School of Optometry and Vision Science, University of New South Wales Sydney, Sydney, NSW, Australia; ^6^Faculty of Medicine and Health Technology, Tampere University, Tampere, Finland; ^7^Expert Microbiology Unit, Department of Health Security, Finnish Institute for Health and Welfare, Helsinki, Finland; ^8^Finnish Food Authority, Seinäjoki, Finland

**Keywords:** systematic review, antimicrobial resistance, wastewater-based epidemiology, clinical surveillance, wastewater surveillance

## Abstract

Infectious diseases caused by antibiotic-resistant bacterial (ARB) pathogens are a serious threat to human and animal health. The active surveillance of ARB using an integrated one-health approach can help to reduce the emergence and spread of ARB, reduce the associated economic impact, and guide antimicrobial stewardship programs. Wastewater surveillance (WWS) of ARB provides composite samples for a total population, with easy access to the mixed community microbiome. This concept is emerging rapidly, but the clinical utility, sensitivity, and uniformity of WWS of ARB remain poorly understood especially in relation to clinical evidence in sewershed communities. Here, we systematically searched the literature to identify studies that have compared findings from WWS of ARB and antibiotic resistance genes (ARG) with clinical evidence in parallel, thereby evaluating how likely WWS of ARB and ARG can relate to the clinical cases in communities. Initially, 2,235 articles were obtained using the primary search keywords, and 1,219 articles remained after de-duplication. Among these, 35 articles fulfilled the search criteria, and an additional 13 relevant articles were included by searching references in the primary literature. Among the 48 included papers, 34 studies used a culture-based method, followed by 11 metagenomics, and three PCR-based methods. A total of 28 out of 48 included studies were conducted at the single sewershed level, eight studies involved several countries, seven studies were conducted at national or regional scales, and five at hospital levels. Our review revealed that the performance of WWS of ARB pathogens has been evaluated more frequently for *Escherichia coli, Enterococcus* spp., and other members of the family *Enterobacteriaceae*, but has not been uniformly tested for all ARB pathogens. Many wastewater-based ARB studies comparing the findings with clinical evidence were conducted to evaluate the public health risk but not to relate with clinical evidence and to evaluate the performance of WWS of ARB. Indeed, relating WWS of ARB with clinical evidence in a sewershed is not straightforward, as the source of ARB in wastewater cannot be only from symptomatic human individuals but can also be from asymptomatic carriers as well as from animal sources. Further, the varying fates of each bacterial species and ARG within the sewerage make the aim of connecting WWS of ARB with clinical evidence more complicated. Therefore, future studies evaluating the performance of many AMR pathogens and their genes for WWS one by one can make the process simpler and the interpretation of results easier.

## Introduction

Antimicrobial resistance (AMR) surveillance collects information on the geographical and seasonal patterns and incidence of AMR, as well as evidence of new or rare resistance traits and emerging trends. Such information is important for mitigating AMR infections, prioritizing actions to be taken, evaluating the impacts of earlier interventions, informing empirical treatment guidelines, reducing overall adverse impacts, and developing new antimicrobial drugs (Masterton, 2008; Tacconelli et al., 2018a). Surveillance information benefits policymakers, the healthcare sector, medicine developers, and scientific communities.

Currently, many surveillance mechanisms and institutions operate in AMR surveillance at national, regional, and global levels (Tacconelli et al., 2018b). Among them, the Global Antimicrobial Resistance and Use Surveillance System (GLASS) of the World Health Organization (WHO) works at the global level with the major aim of a global collaborative effort to standardize AMR surveillance and strengthen knowledge through extensive research. In the European Union (EU), two surveillance networks exist; these are the European Antimicrobial Resistance Surveillance Network (EARS-Net) and the European Surveillance of Antimicrobial Consumption Network (ESAC-Net) covering 30 countries in the European economic area (Tacconelli et al., 2018b). In addition, the central Asian and European Surveillance of Antimicrobial Resistance (CAESAR) network includes 19 countries, primarily in eastern Europe and central Asia. Tacconelli et al. (2018b) listed about 38 national and regional AMR surveillance institutions and mechanisms in Europe.

Many of the national and international AMR surveillance initiatives focus on a few pathogens in clinical settings. However, such clinical isolate-based surveillance requires large numbers of patients for getting meaningful information and is based on passive reporting of phenotypic laboratory results. Although the clinical isolate-based approach can provide information on multidrug-resistant pathogens, this approach is biased, as it only includes diseases with clinical signs and symptoms and hospitalized patients but does not consider AMR in the commensal microbiota of healthy individuals. Therefore, it cannot represent the prevalence of AMR in an overall population (WHO, 2018; Aarestrup and Woolhouse, 2020; ECDC, 2020).

Wastewater surveillance (WWS), i.e., monitoring municipal sewage, can be a cost-effective approach for predicting the occurrence of antibiotic-resistant bacteria (ARB) and respective genes at the population level (Ng et al., 2017; Khan et al., 2018; Hutinel et al., 2019; Pärnänen et al., 2019; Aarestrup and Woolhouse, 2020; Huijbers et al., 2020; Sims and Kasprzyk-Hordern, 2020; Blaak et al., 2021). From a surveillance point of view, municipal sewage can be good material for ARB monitoring, as it comprises bacterial biomass from all ARB, including pathogens from the entire population of a community, contributed from the early stage of colonization to the different stages of infections (symptomatic, asymptomatic, pre-symptomatic, and post-symptomatic), contributing through feces, urine, nasal mucus, skin, and sputum to sewage from households, hospitals, and nursing homes (Diemert and Yan, 2019; Flach et al., 2021). As this approach does not collect samples on an individual level, it has minimal legal and ethical challenges and individual privacy concerns (Auguet et al., 2021). Furthermore, WWS can provide an opportunity for real-time monitoring or even an early prediction tool for future infection outbreaks, and a tool for observing the status of multidrug-resistant (MDR) pathogens at the population level (Flach et al., 2021).

Multiple review papers have been published on WWS of AMR with differing focuses, such as methodologies (Karkman et al., 2018; Miłobedzka et al., 2022), bioinformatics pipelines (Hendriksen et al., 2019a), opinion reviews (Aarestrup and Woolhouse, 2020; Pruden et al., 2021), critical reviews (Rizzo et al., 2013; Fahrenfeld and Bisceglia, 2016; Huijbers et al., 2019), challenges with limitations (Larsson et al., 2018), and systematic reviews with meta-analyses (Pazda et al., 2019; Sims and Kasprzyk-Hordern, 2020; Zaatout et al., 2021; Chau et al., 2022). However, the possible contribution of ARB from non-human sources, factors affecting the fate and distribution of ARB pathogens in the sewerage network, possible selective proliferation due to various biocides, and possible horizontal gene transfer (HGT) in sewerage networks are not sufficiently known. In this systematic review, we examine closely such issues and compare WWS of ARB with clinical surveillance. Furthermore, we evaluate the diversity of targets and methodology applied in the reviewed literature.

## Conceptual and theoretical framework

### What is antimicrobial resistance?

Antimicrobial resistance (AMR) is the ability of an organism to resist the effect of an antimicrobial compound. The AMR capacity of the organism can be intrinsic, i.e., due to the structural properties of an organism, or also due to acquired resistance determinants, such as genes encoding enzymes targeting the antimicrobial molecule. The dissemination of such AMR in the environment is primarily caused by three mechanisms: (a) horizontal gene transfer (HGT) of antimicrobial resistance genes via mobile genetic elements such as plasmids, transposons, and integrons, (b) genetic mutations, and (c) subsequent vertical gene transfer of these mutations (Wellington et al., 2013; Berendonk et al., 2015; Martinez and Baquero, 2017). Often, AMR and antibiotic resistance are used synonymously, but AMR is a broader term that includes all microorganisms, while antibiotic resistance covers exclusively bacteria. The clinical definition of antibiotic resistance is based on the likelihood that the treatment of an infection with a given bacterium would not result in clinical success. Susceptibility testing classifies clinical bacteria as “susceptible,” “intermediate,” or “resistant” to an antibiotic. This classification relies on the growth of the bacterium *in vitro* in different antibiotic concentrations, referred to as “breakpoints” for clinical treatment purposes (European Committee on Antimicrobial Susceptibility Testing, 2022). This definition is primarily used by clinicians for prescribing antibiotics. In this approach, bacterial isolates are cultured on growth media supplemented with a certain concentration of antibiotics to determine the minimal inhibitory concentration (MIC) and phenotypic resistance (European Committee on Antimicrobial Susceptibility Testing, 2022).

Environmental bacteria can potentially transfer resistance genes to clinical pathogens, so the prevalence of ARB from environmental sources deserves particular attention. The clinical definition does not distinguish between bacteria with and without phenotypically expressed resistance mechanisms. Furthermore, this definition does not apply to non-clinical bacteria and resistance against antimicrobial compounds that are not used for therapeutic purposes (Martínez et al., 2015). In this case, epidemiological cut-off (ECOFF) values are determined. The ECOFF value is independent of therapeutic efficiency and separates populations with acquired resistance mechanisms (non-wild types) from wild-type populations that have no resistance within a given taxonomic group. Here, the upper limit of inhibition concentration for a wild-type population of a species is determined and used for comparison with a resistant type. The ECOFF-based definition is based on the screening of a large number of isolates of a given bacterial population and the identification of resistance ones that have higher minimal inhibitory concentrations than the bulk of the population. This definition can be used for all types of microorganisms and all types of biocidal compounds for which the clinical definition of resistance does not apply (Martínez et al., 2015).

Cultivation-independent PCR-based methods consider bacteria as antibiotic-resistant if they carry resistance genes (Martinez and Baquero, 2017). The molecular analysis-based definition also covers non-culturable bacteria, with direct extraction of environmental DNA from wastewater and monitoring of resistance genes using a PCR-based method or high-throughput shotgun metagenomics (Karkman et al., 2016; Buelow et al., 2018). However, this approach cannot link antibiotic resistance genes (ARGs) with the susceptibility level of clinical pathogens (susceptible, intermediate, or resistant), as a susceptibility test is not possible with this approach. The molecular analysis-based definition has provided evidence that ARB can be found everywhere, even in drinking water produced from groundwater (Tiwari et al., 2022c).

### Antimicrobial resistance as a global threat

Antimicrobial resistance is a serious threat to human, animal, and environmental health (Zhang et al., 2021). Based on the One Health perspective, contaminants from among these three compartments can easily jump to the others through food and water and cause effects. Currently, the world is passing through a serious silent pandemic of AMR (World Health Organization, 2021). The WHO declared AMR as one of the top ten global health threats facing humanity, requiring urgent action to achieve sustainable development goals (World Health Organization, 2021). In 2019, AMR globally caused about 1.27 million deaths (Muray et al., 2022), and millions of people suffered due to prolonged illness and hospitalization. If appropriate action against AMR proliferation is not urgently taken, human casualties could accelerate to ten million per year by 2050 (O’Neill, 2016).

Globally, the six leading ARB pathogens, comprising *Escherichia coli, Staphylococcus aureus, Klebsiella pneumoniae, Streptococcus pneumoniae, Acinetobacter baumannii*, and *Pseudomonas aeruginosa*, account for more than 71% of all deaths caused by AMR pathogens (Muray et al., 2022). The characteristics of major ARB pathogens, including their physiological characteristics, major infection, treatment, and major antibiotic resistance, are summarized in Table 1. From a nosocomial infection risk perspective, ESKAPE, an acronym representing the scientific names of six highly virulent ARB pathogens (*Enterococcus faecium, S. aureus, K. pneumoniae, A. baumannii, P. aeruginosa*, and *Enterobacter* species), are critical for human health, as they often exhibit multidrug resistance (Table 1). Furthermore, based on the urgency of developing alternative medicines, WHO has listed 12 bacterial pathogens as potentially threatening human health (Tacconelli et al., 2018a). This list mainly includes Gram-negative bacteria commonly associated with hospital and/or community-acquired infections (Table 1). WHO has divided these ARB pathogens into three categories, namely critical, high, and medium priority, based on their impact on human health and the urgency of developing new medicines to treat them (Davies and Simeon, 2017; World Health Organization, 2021). Two carbapenem-resistant species, *A. baumannii* and *P. aeruginosa*, with *Enterobacteriaceae*, which are carbapenem-resistant, extended-spectrum beta-lactamase (ESBL)-producing pathogens, have been placed in the critical (priority one) category (Davies and Simeon, 2017). Vancomycin-resistant *E. faecium*, methicillin-resistant, vancomycin-intermediate and resistant *S. aureus*, clarithromycin-resistant *Helicobacter pylori*, fluoroquinolone-resistant *Campylobacter* spp., and *Salmonella* spp., and cephalosporin-resistant and fluoroquinolone-resistant *Neisseria gonorrhoeae* are placed in the high priority (priority two) category (Davies and Simeon, 2017). Meanwhile, penicillin-non-susceptible *S. pneumoniae*, ampicillin-resistant *Haemophilus influenzae*, and fluoroquinolone-resistant *Shigella* spp. are placed in the medium priority (priority three) category (Davies and Simeon, 2017). Moreover, the European surveillance system EARS-Net places importance on the surveillance of the following eight ARB pathogens: *E. coli, K. pneumoniae, P. aeruginosa, Acinetobacter* species, *S. pneumoniae, S. aureus, E. faecalis*, and *E. faecium* (Cristea, 2016; ECDC, 2020).

**TABLE 1:** Characteristics of major antibiotic resistant bacterial pathogens.

Pathogens	Physiological characteristics	Major infection	Treatment	Major antibiotics possessing resistance
Pathogenic *Escherichia* *coli*^μ,^ *^γ,^* ^θ^	Gram-negative facultative anaerobes, common gut bacteria.	Gastroenteritis, diarrhea, UTI, neonatal meningitis, Crohn’s disease, hemorrhagic colitis, hemolytic-uremic syndrome, & sepsis. Mostly transfer from the fecal–oral route.	Macrolide (azithromycin), cephalosporin (cefixime, ceftriaxone), fluoroquinolones (ciprofloxacin), tetracycline (doxycycline), fosfomycin, levofloxacin, phenicol, rifaximin, sulphonamides (sulfamethoxazole), trimethoprim.	Resistance to cefotaxime, fluoroquinolone, colistin, and carbapenem.
*Staphylococcus aureus*^μ,^ *^β,^* ^δ, θ^	Gram-positive *Bacillota*, facultative anaerobes, commensal & opportunistic pathogens frequently found in URT, nostrils, & the skin.	Skin infections include abscesses, RTI, food poisoning, surgical wound infection, bacteremia, or sepsis when bacteria spread to the bloodstream, and osteomyelitis	Cephalosporins, lincomycin (clindamycin), cotrimoxazole, erythromycin, fusidic acid, lincosamides (lincomycin), oxazolidinones (linezolid), penicillin (methicillin), carboxylic acid (mupirocin), rifampicin, glycopeptide (vancomycin).	Resistance to methicillin and vancomycin.
*Klebsiella pneumoniae*^μ,^ *^β, γ,^* ^θ^	Gram-negative, facultative-anaerobic, normal microbiota of mouth, skin, and intestines.	Pneumonia, UTI, infection in the lower biliary tract, upper RTI, surgical wound infection, thrombophlebitis, cholecystitis, diarrhea, osteomyelitis, meningitis, bacteremia, & sepsis.	Aminoglycosides, aztreonam, carbapenems (imipenem), cephalosporins (3^rd^ & 4^th^ Gen), penicillin (piperacillin), fluoroquinolones (quinolones), rifampin, sulfonamides, tazobactam, tetracycline.	Resistance to colistin, different betalactam antibiotics including carbapenem.
*Streptococcus pneumoniae* ^μ, θ^	Gram-positive, alpha-hemolytic (under aerobic conditions) or beta-hemolytic (under anaerobic conditions), aerotolerant anaerobic. Normal microbiota in the respiratory tract, sinuses, and nasal cavity.	Neonatal infections, bronchitis, rhinitis, acute sinusitis, otitis media, conjunctivitis, meningitis, sepsis, osteomyelitis, septic arthritis, endocarditis, peritonitis, pericarditis, cellulitis, and brain abscess.	Carbapenem, cephalosporins, lincomycin (clindamycin), glycopeptides, lincosomides, lipopeptides, macrolides, aminoglycosides, penicillin, rifampin, sulphonamides (sulfamethoxazole), tetracyclines, trimethoprim, vaccination.	Resistance to penicillin.
*Acinetobacter baumannii*^μ,^ *^β, γ,^* ^θ^	Gram-negative, mostly soil bacteria, and often reported from hospitals.	Opportunistic pathogen, nosocomial infection. Bloodstream infections, UTI, pneumonia.	Aminoglycosides, carbapenems, fosfomycin, piperacillin, polymyxin, rifampin, sulbactam, tazobactam, tigecycline, glycopeptide (vancomycin).	Resistance to carbapenem, cephalosporin, fluoroquinolone, trimethoprim, sulfamethoxazole, colistin.
*Pseudomonas aeruginosa*^μ,^ *^β, γ,^* ^θ^	Gram-negative, strictly aerobic but can grow anaerobically in the presence of nitrate. Ubiquitous, in soil, water, skin microbiota, most man-made environments, & hospital environments.	One of the most virulent pathogens, nosocomial infection, opportunistic pathogen, blood infection, pneumonia, surgical wound infection, Infect critical body organs, such as lungs, kidneys, & UTIs.	Aminoglycosides, carbapenems, cephalosporins (3rd & 4th Gen), fluoroquinolones (quinolones), carbapenems (imipenem, meropenem), penicillin.	Resistance to multiple drugs, carbapenems, fluoroquinolones, aminoglycosides, and cephalosporins (3^rd^ & 4^th^ Gen).
*Enterococcus faecium* ^β, δ, θ^	Gram-positive facultative anaerobic cocci, normal gut microbiota.	Nosocomial infections. UTI, intra-abdominal infections, bacteremia, meningitis, infective endocarditis.	Cephalosporins, penicillin (ampicillin), glycopeptide (vancomycin).	Resistance to ampicillin, vancomycin.
*Enterobacter* species*^β, γ^*	Gram-negative, facultative anaerobic rods. Predominant natural habitat (soil, water), Normal gut microbiota.	Opportunistic pathogens (*E. cloacae*, *E. aerogenes*, *E. gergoviae*, & *E. agglomerans*), can cause eye & skin infections, meningitis, bacteremia, pneumonia, & UTIs. *E. cloacae* & *E. aerogenes* cause nosocomial infections.	Aminoglycoside, fluoroquinolone, cephalosporin, & carbapenems (imipenem).	Beta-lactam, including carbapenems and multidrug-resistant.
*Serratia* spp.^δ^	Gram-negative, facultative anaerobic rods, *Enterobacteriaceae* common in the environment, but not common human fecal microbiota.	Opportunistic pathogen, nosocomial infections of the bloodstream, lower respiratory tract, urinary tract, surgical wounds, skin & soft tissues.	Aminoglycoside (amikacin, gentamicin), antipseudomonal beta-lactam, cephalosporin (cefepime, ceftazidime), fluoroquinolones (quinolones), tobramycin.	Resistance to cefotaxime, carbapenem, and many other ß-lactams.
*Proteus* spp.^δ^	Gram-negative, facultative anaerobic, *Enterobacteriaceae*, commensal GI bacteria.	UTI, RTI, kidney stones, sepsis, diarrhea, and other infections	Penicillin (ampicillin), aztreonam, cephalosporin (ceftriaxone), fluoroquinolone (ciprofloxacin), aminoglycoside (gentamicin), fluoroquinolones (quinolones), sulphonamides (sulfamethoxazole), diaminopyrimidines (trimethoprim).	Resistance to carbapenem & many other ß-lactams.
*Helicobacter pylori* ^δ^	Gram-negative, microaerophilic, spiral (helical) bacterium usually found in the stomach	Stomach infection, peptic ulcers	Amoxicillin, macrolide (azithromycin, clarithromycin), nitroimidazole (metronidazole, tinidazole), tetracycline.	Resistance to metronidazole & clarithromycin
*Campylobacter* spp.^δ^	Gram-negative spiral-shaped rod, microaerophilic, cannot tolerate drying, zoonotic bacteria common in warm-blooded animals & widely prevalent in food animals & pets.	Food or waterborne infection, gastrointestinal disease causes severe abdominal pain, watery and/or bloody diarrhea, nausea, headache, & fever, joint inflammation, or Guillain-Barré neurological syndrome.	Macrolide (azithromycin, erythromycin), fluoroquinolones (ciprofloxacin, quinolone), tetracycline.	Resistance to fluoroquinolone, ciprofloxacin, tetracycline, quinolone.
*Salmonella* spp.^δ^	Gram-negative rods, ubiquitous, survive long in the environment. Some serotypes are host specific, but all can be human pathogenic and zoonotic pathogens.	Diarrhea, fever, abdominal pain, nausea, & vomiting. Symptoms are relatively mild but can be severe or life-threatening in young & elderly patients.	Fluoroquinolones and macrolide (azithromycin).	Resistance to fluoroquinolone, sulphonamide, ampicillin, tetracycline, ciprofloxacin, cefotaxime.
*Neisseria gonorrhoeae* ^δ^	Gram-negative, oxidase-positive diplococcus.	Sexually transmitted infection. In men: urethritis, possible epididymitis, urethral stricture, & infertility. In women, usually asymptomatic, may lead to pelvic inflammatory disease, ectopic pregnancies, & infertility.	Cephalosporin (cefixime, ceftriaxone), fluoroquinolone, spectinomycin, macrolide (azithromycin), aminoglycoside (gentamicin, kanamycin).	Resistance to cephalosporin, fluoroquinolone, sulphonamide, penicillin, tetracycline, and macrolide.
*Haemophilus influenzae* ^ε^	Gram-negative, facultatively anaerobic	Meningitis, pneumonia, otitis media	Penicillin (ampicillin), sulbactam, fluoroquinolones, cephalosporin (ceftriaxone, cefotaxime), and macrolides.	Resistance to ampicillin, penicillin, macrolide, fluoroquinolone, including many other beta-lactams.
*Shigella* spp.^ε^	Gram-negative, facultative anaerobic *Enterobacteriaceae*, intracellular pathogens, specific to human or primate hosts.	Shigellosis, diarrhea (sometimes bloody), fever, and stomach cramps	Sulfonamides, tetracyclines, penicillin (ampicillin), trimethoprim, fluoroquinolone, sulphonamides (sulfamethoxazole).	Resistance to fluoroquinolone, sulfonamides, tetracyclines, ampicillin, trimethoprim-sulfamethoxazole.

Pathogens with **μ** are among six major global fatal manifestations, Pathogens with **β** are ESKAPE nosocomial pathogens, **γ** denotes WHO critical priority pathogens [40], δ are WHO high priority [40], ε are WHO medium priority [40], θ are EARS-NeT monitoring pathogens [4, 41], URT = upper respiratory tract, HAI = hospital acquired infection, UTI = urinary tract infection.

### Antibiotic-resistant bacterial from human shedding and their fate and decay in sewerage systems

The consumption of antibiotics creates selective pressure on bacterial communities in the human body, including those of the gut, skin, and blood (Talebi et al., 2008; Ruppé et al., 2013; Hu et al., 2014; Drieux et al., 2016). Bacteria in the human gut may also acquire antimicrobial agents from many secondary sources, such as the consumption of contaminated food and water (Hu et al., 2014; Casals-Pascual et al., 2018; Bich et al., 2019; Tiwari et al., 2022c). Moreover, the human gut can form a perfect ecological environment for the HGT of ARGs via mobile genetic elements such as plasmids, transposons, and integrons, genetic mutations, and the subsequent vertical gene transfer of these mutations (Shoemaker et al., 2001; Schjørring and Krogfelt, 2011; Penders et al., 2013). Therefore, human communities can be an important reservoir of ARB, continuously contributing to the sewerage system (Hu et al., 2014; Pieri et al., 2020).

Despite the high diversity of gut bacterial communities, two major phyla, *Bacteroidota* (earlier commonly known as *Bacteroidetes*) and *Bacillota* (earlier *Firmicutes*), and two minor phyla, *Actinomycetota* (earlier *Actinobacteria*) and *Pseudomonadota* (earlier *Proteobacteria*) are the most dominant in the human gut microbiota (The Human Microbiome Project Consortium, 2012; Casals-Pascual et al., 2018). However, the diversity of gut bacterial communities can be affected by many factors, such as diet, health status, age, and medicine consumption, resulting in variation from one person to another. The ambient ecological conditions of gut microbes consist of a mesophilic temperature (∼37 °C), an environment rich in nutrients, a high salt and bile concentration, anoxic conditions, and high osmotic pressure. Regarding ARB communities in the human gut, the majority of bacteria (∼80%) are obligatory anaerobic and mesophilic (∼90%) and only a few (∼11%) are facultative anaerobic species (Hu et al., 2014). As soon as bacteria are excreted from such an environment to the sewerage network, they face tremendous ecological pressures, including a drop in temperature, osmotic pressure, nutrient and bile concentrations, a higher oxygen concentration, probable exposure to light, biological interaction (predation and phage effect), and exposure to other biocides such as chlorine and detergents. In addition to gut bacteria transferred through the feces, additional bacteria reach sewerage systems through urine, nasal mucus, sputum, and skin during baths, and many environmental bacteria from water systems and also through run-off waters. However, different bacterial species from the human body can have variations in fate and survivability in the sewerage network (Kizny Gordon et al., 2017; Casals-Pascual et al., 2018). For example, *Bacteroidota*, which are strict anaerobes, may die more rapidly than *Bacillota* and *Pseudomonadota*, which are facultative anaerobes (McLellan and Eren, 2014). Therefore, the likelihood of detecting ARB in sewage can be affected by the survival rate of such bacterial communities in sewerage systems.

Furthermore, wastewater is rich in a wide range of antimicrobial agents, such as partially metabolized antibiotics, detergents, heavy metals, and biocides. These compounds together can exert selective pressure on the proliferation of ARB by suppressing susceptible communities (Wellington et al., 2013; Berendonk et al., 2015; Martinez and Baquero, 2017). The wastewater distribution system is a nutrient-rich environment and that can be an ideal niche for the proliferation of certain environmental bacteria (Pilmis et al., 2020). The high microbial load in the distribution system can be an ideal situation for the proliferation of ARB through the process of HGT of ARGs. Furthermore, biofilm formation is one of the basic mechanisms allowing bacteria to flourish, and many bacterial groups can form biofilms in sewerage networks, better protecting them from environmental stresses (Auguet et al., 2017).

Healthcare settings such as hospitals and nursing homes can act as point sources of contamination of municipal sewerage systems with ARB and ARGs (Rodriguez-mozaz et al., 2014; Khan et al., 2019; Perry et al., 2021). Greater quantities and different classes of antibiotics are being used in healthcare settings when compared to communities (ECDC, 2020). Therefore, sewage from healthcare settings more represents clinical patients than a whole community. Furthermore, the sewage from healthcare settings can be rich in discarded unused and partially metabolized medicines excreted through the feces and urine of clinical patients, creating extra selective pressure for ARB in sewerage networks (Rodriguez-mozaz et al., 2014).

## Methodology

**FIGURE 1:**
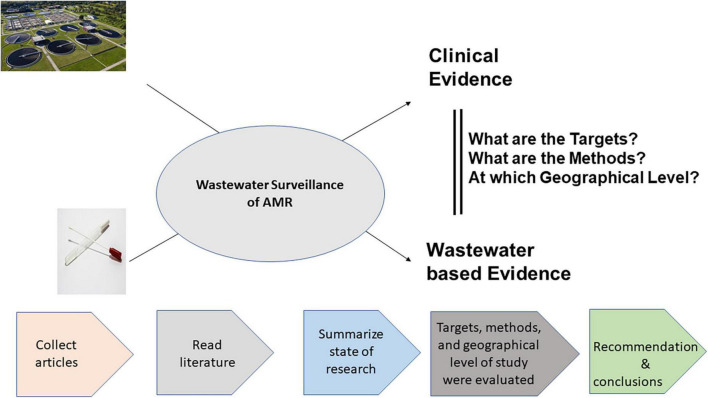
Graphical representation of study design and summary.

The literature was searched on 16.01.2022 by using the electronic databases PubMed, Science Direct, Google Scholar, Web of Science, and Scopus. The search was limited to peer-reviewed published articles in the English language without any restriction on the years of publication. The search term was improved while conducting a trial search and looking for other relevant terms within each concept from the retrieved papers. During the literature search, the Boolean search technique was employed by combining the keywords using “AND,” and “OR.” The Preferred Reporting Items for Systematic Reviews and Meta-Analysis (PRISMA) guideline was followed during the literature search (Moher et al., 2009). Searches were directed toward review objectives, using search keywords targeting the title of publications (Supplementary material 1) initially, all obtained articles were saved in the reference management tool EndNote (Clarivite Analytics). Then, duplicate publications obtained from various search engines were removed by using the “Find Duplicates” function. A detailed literature review flow chart and inclusion/exclusion criteria are presented in Supplementary material 2. Titles of all remaining articles were screened, and the potential papers were listed. Then, after that, the titles and abstracts of the remaining articles were manually screened to find appropriate studies based on the research question. Our outcome included a wide variety of literature related to wastewater surveillance and fecal sample analysis for ARB that compared the findings with clinical evidence and indirectly attempted to evaluate the usefulness of WWS. In such comparisons, clinical evidence was obtained from primary studies (active surveillance) and secondary literature, including clinical surveillance reports or governmental reports. Studies on WWS of ARB in which no comparison was made with clinical evidence and studies that did not generate any primary data including reviews, systematic reviews, literature reviews, perspectives, or meta-analyses papers were excluded. Additional relevant literature was also screened from the reference lists (based on screening titles) of the primary literature i.e., articles collected through the key phrase searches. Then, studies were grouped into different geographical levels such as intercontinental, international (more than one continent or country), national and regional (more than two sewershed areas), single sewershed, and hospital levels (Figure 1).

## Results

Out of 2,235 articles obtained with the search for keywords, a total of 1219 was retained after deduplication. After screening the topics and abstracts, a total of 127 articles were retained. After reading the abstract of these articles, 35 of them met our inclusion criteria, i.e., studies that monitored ARB or related genes in wastewater and compared the findings with clinical evidence. The excluded papers were mainly surveillance papers that did not compare the results with clinical evidence. Furthermore, 13 relevant studies were obtained by screening the reference lists of other searched articles. These studies were missed in the primary literature search due to having titles lacking the primary search keywords. Hence, 48 studies were included in this systematic review, of which 28 were conducted at the single sewershed level (i.e., single sewer catchment), nine studies were conducted at the international levels (out of nine, seven studies were conducted at the intercontinental level), and seven studies were conducted at the national or regional level (at least two or more cities or more sewerage networks), and five studies were conducted at the hospital level (Supplementary Tables 1, 5). The majority of studies (31 out of 48 studies) were conducted in Europe or led by European research institutes, followed by Asia (10/48 studies), North America (4/48 studies), and one study each in Africa, South America, and Australia.

**FIGURE 2:**
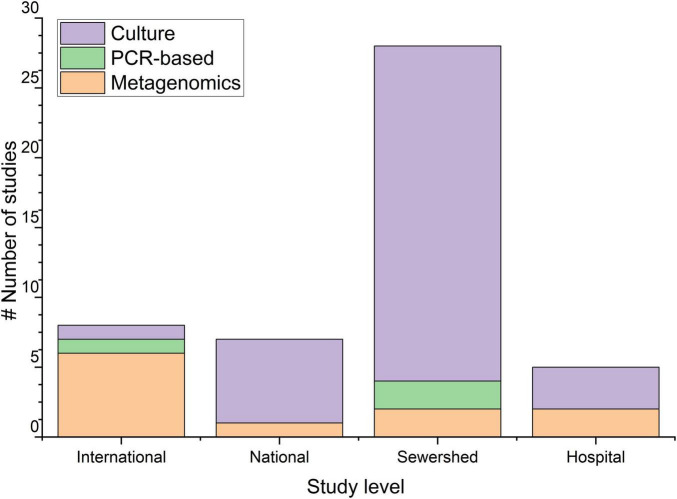
The number of studies conducted at different study levels. International, studies conducted in more than two countries, National/Regional, studies conducted in more than one wastewater treatment plant inside a country, Sewershed, studies conducted within a sewershed, Hospital, studies conducted in a hospital.

**FIGURE 3:**
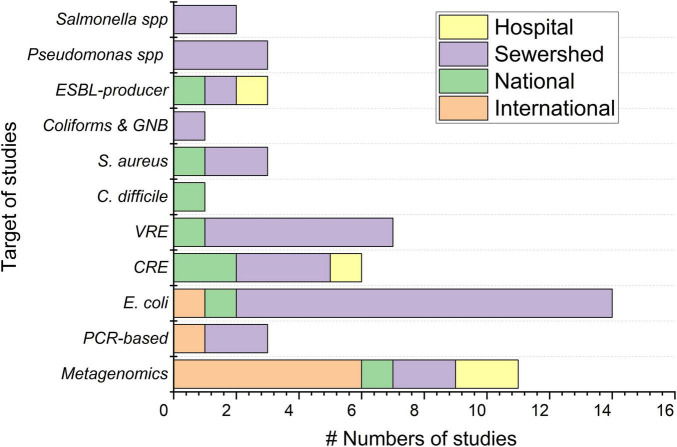
Number of studies using different ARB targets. International, studies conducted in more than two countries (pooled intercontinental within international), National/Regional, studies conducted in more than one WWTP inside a country; Sewershed, studies conducted within a sewershed*;* Hospital, studies conducted at the hospital level; CRE, carbapenem-resistant *Enterobacteriaceae*; VRE, vancomycin-resistant enterococci, ESBL, extended-spectrum beta-lactamase; GNB, gram-negative bacteria.

Regarding the methodologies applied, the culture-based method was most frequently used (75%; 36/48 of the total) (Figure 2). The majority of these studies using culture-based methods were carried out at a single sewershed level (*n* = 25), then followed by the national level (*n* = 6), hospital level (*n* = 4), and international level (*n* = 1), but not used in intercontinental level (*n* = 0). Among the studies employing culture-based methods, fourteen studies targeted *E. coli* (Jakobsen et al., 2008; Yang et al., 2009; Reinthaler et al., 2013; Zarfel et al., 2013; Jørgensen et al., 2017; Ojer-usoz et al., 2017; Hutinel et al., 2019; Paulshus et al., 2019; Raven et al., 2019; Adator et al., 2020; Huijbers et al., 2020; Kolokotsa and Leotsinidis, 2020; Urase et al., 2020), seven targeted vancomycin-resistant *Enterococcus* spp. (Talebi et al., 2008; Saifi et al., 2009; Oravcova et al., 2017; Gouliouris et al., 2019; Haghi et al., 2019; Kolokotsa and Leotsinidis, 2020; Zaheer et al., 2020), and six studies targeted carbapenem-resistant *Enterobacteriaceae* (CRE) (Meir-Gruber et al., 2016; Adator et al., 2020; Urase et al., 2020; Blaak et al., 2021; Flach et al., 2021; Tiwari et al., 2022e). Other studies targeted ESBL-producing bacteria (Drieux et al., 2016; Urase et al., 2020; Rodríguez et al., 2021), *Pseudomonas* spp. (Golle et al., 2017; Abduljabbar and Aljanaby, 2018; Kolokotsa and Leotsinidis, 2020), *Staphylococcus aureus* (Rahimi and Bouzari, 2015; Meir-Gruber et al., 2016; Kolokotsa and Leotsinidis, 2020), *Salmonella* spp. (Pignato et al., 2010; Diemert and Yan, 2019), *Clostridioides difficile* (earlier *Clostridium difficile*) (Moradigaravand et al., 2018), coliform group bacteria (Khan et al., 2019), and *Campylobacter* spp. (Mourkas et al., 2019) (Figure 3). These studies mostly reported the seasonal variation and detection frequency of the ARB targets (Supplementary Tables 1, 5). Two other studies conducted at the single sewershed level used PCR-based methods (Bich et al., 2019; Mtetwa et al., 2021), and one study used shotgun metagenomics (Majeed et al., 2021). Most of these studies compared wastewater-based findings with clinical isolates collected at the single hospital level, while some studies also collected clinical isolates from human patients, and others compared the findings with government reports and secondary literature (Supplementary Table 3).

Except for one study, studies conducted at the international level used shotgun metagenomics or high-throughput PCR and reported the geographic “hot spots” of ARB (Hendriksen et al., 2019b; Pärnänen et al., 2019; Riquelme et al., 2021). These studies reported a relative abundance of ARB, and resistance genes. One technological limitation of these molecular methods is that they cannot elucidate the pathogenicity of the ARB pathogens as detecting ARG is not sufficient to determine whether it is hosted by a pathogen or a commensal or an environmental bacterium. Most of these studies reported that ARB and related genes were more prevalent in regions with higher antibiotic consumption, poor sanitation, and low socioeconomic status. These studies compared wastewater-based results with World Bank’s Human Development Index (HDI) data (Hendriksen et al., 2019b; Karkman et al., 2020) and cases reported in EARS-Net (Pärnänen et al., 2019; Huijbers et al., 2020; Karkman et al., 2020). More than half of the metagenomics studies (54.5%, 6/11 studies) were conducted at the intercontinental and international levels.

Most studies carried out at the national level reported temporal and/or spatial hotspots of ARB pathogens. In general, these studies compared wastewater-based findings with clinical isolates collected at the national level (Reinthaler et al., 2013; Moradigaravand et al., 2018; Gouliouris et al., 2019), areas with and without hospitals (Blaak et al., 2021), government-reported data, and secondary literature (Meir-Gruber et al., 2016; Su et al., 2017; Urase et al., 2020). Regarding studies conducted at the hospital level, four studies used culture-based methods, and two studies used shotgun metagenomics to indicate a possible relationship between ARB pathogens collected in wastewater and clinical isolates.

Regarding the comparison of wastewater-based and clinical findings, most studies compared the findings from wastewater with independent clinical data from hospitals, antibiotic consumption patterns, earlier published peer-reviewed clinical papers from the study area, and official clinical surveillance databases such as EARS-Net. Most of these studies reported concordance between WWS of ARB isolates and ARGs with clinical evidence. One study reported a relationship between WWS of ARGs with antibiotic consumption in a community (Pärnänen et al., 2019).

## Discussion

Wastewater surveillance (WWS) is an emerging approach for monitoring ARB at all global, national, seweshed, and hospital population levels. WWS of ARB helps to determine the existing situation and identify spatial and temporal trends. Theoretically, it can provide evidence of resistance traits in bacteria and can act as an early prediction tool at a community level. Even before the first symptoms, i.e., immediately after exposure or during the early stages of colonization, an individual can start to shed pathogen biomarkers to the municipal sewage systems through various body excretions (feces, urine, nasal mucus, and sputum) and thus make an early detection possible with WWS. In contrast, clinical surveillance usually takes many days, due to multiple different steps and stages involved, such as exposure to pathogens, colonization, clinical symptoms, testing, detection, sample collection, analysis, and reporting (Thakali et al., 2022; Tiwari et al., 2022a). Even, all symptomatic individuals may not seek clinical testing. The currently used clinical isolate-based surveillance approach reports annually and is based on passive reporting, which is not effective for proactive ARB pathogen management or outbreak mitigation actions (Diemert and Yan, 2019). Therefore, WWS can provide evidence for possible outbreaks before any evidence is available from clinical surveillance. But in the environment, dilution or die-off of ARB pathogens due to adverse conditions within the environment may push them below the radar, which may be problematic for WWS and may require particular approaches such as enrichment procedures to keep a descent level for surveillance. Furthermore, often colonization and infection of individuals do not reach an outbreak level, and, when ARGs are concerned, they may not be exclusively hosted by pathogenic bacteria. Many of them can enter the sewage system through commensal bacteria and through non-human sources. Therefore, WWS of ARB can have a significant false positive rate for ARB pathogen outbreaks. However, from the perspective of prevention of an outbreak, a false positive alarm that provides time for alerting can be better than having a false negative alarm.

Wastewater surveillance of ARB has not yet been fully developed and there is a lot of work that needs to be done before using the approach as a reliable early warning tool for future ARB pathogen outbreaks. For example, understanding the prevalence of each ARB pathogen and related genes in different environments, such as non-infected healthy individuals and the sewage system of clinical settings (hospitals and nursing homes) during non-outbreak (normal) situations is important. In addition, it is crucial to understand the fate and transmission pathways of pathogens in the sewage system. The detection of ARB and related genes in sewage does not automatically imply an “outbreak”. As mentioned earlier, there are many steps (colonization and infection) before an actual outbreak. Many colonization events do not reach to infection status and many of the detected ARGs can be from an animal host, symbiotic bacteria, and environmental sources.

The current systematic review revealed that not all clinically relevant ARB pathogen has been equally investigated in wastewater and simultaneously compared with clinical evidence. These types of studies have frequently been conducted on clinically relevant pathogens having a fecal origin, including *E. coli, K. pneumoniae, Enterobacter* species, and *Enterococcus* species, so their load in wastewater could be high enough for detection. These pathogens are among the major global fatal manifestations and are WHO critical priority pathogens with nosocomial transmission risks. However, *S. pneumoniae*, *A. baumannii*, and *P. aeruginosa* are also among the six major global fatal manifestations and WHO critical priority pathogens with nosocomial transmission risks but were not common targets in the articles identified in our literature search. There could be various reasons for this. For example, in the case of *S. pneumoniae*, a normal part of the microbiota in the respiratory tract that mostly causes respiratory infections, they cannot have sufficient load to be detected in wastewater. In the case of *A. baumannii* and *P. aeruginosa*, the low number of studies with these as targets in our review could be due to our literature inclusion criteria. We included studies focusing on wastewater that simultaneously compared the findings with clinical evidence. However, *A. baumannii* and *P. aeruginosa* are of environmental origin and are opportunistic, nosocomial pathogens, frequently reported either from clinical studies or from wastewater (Dharmsthiti and Kuhasuntisuk, 1998; Slekovec et al., 2012; Hrenovic et al., 2016; Higgins et al., 2018; Dekic et al., 2019; Menon et al., 2021) but rarely studied simultaneously with both approaches together. In the same way, *Serratia* spp. (a WHO high-priority pathogen) is also of environmental origin and an opportunistic and nosocomial pathogen that warrants further study in wastewater. Wastewater from healthcare institutes can provide a good sample matrix for the surveillance of nosocomial pathogens such as *A. baumannii, P. aeruginosa*, and *Serratia* spp. Similarly, a sexual transmission agent, *Neisseria gonorrhoeae*, has been reported in wastewater but also deserves more study relating it to clinical evidence. Fecal pathogens such as *Campylobacter* spp., *Salmonella* spp., *Shigella* spp., and *Helicobacter pylori* are potential zoonotic pathogens and are commonly reported in wastewater, but their detection in wastewater may need further validation with human clinical evidence. There could be many other resistant pathogens and genes than those highlighted by the WHO (Zhang et al., 2021). For example, Zhang et al. (2021) presented an ‘omics-based’ framework to evaluate ARG risk considering human-associated-enrichment, gene mobility, and host pathogenicity and classified current and future AMR threats (Zhang et al., 2021). They evaluated the prevalence of “high-risk” (clinically relevant and virulence ARG) in the gut of 1,921 human individuals and reported many new “high-risk” ARG, that are not in the current WHO priority lists (Zhang et al., 2021).

Various factors can affect the detection of a pathogen in sewage, including the site of replication in the host (gastrointestinal (GI) tract, upper respiratory tract, nose, skin, internal organs), the duration of release from the host, the concentration at the source, prevalence of infected individuals, dilution in water (per capita water use, precipitation, or industrial discharge), seasonal factors, and survival in the sewerage system (Sinclair et al., 2008). WWS can be more suitable for pathogens of fecal origin and has the potential to cause GI infections and multiple other infections, such as urinary tract infections, pneumonia, infections of the skin, surgical wounds, and soft tissues, and septicemia, so the target bacterial load in wastewater can be high enough for detection (Sinclair et al., 2008). For example, *E. coli* and *K. pneumoniae* from the *Enterobacteriaceae* family can be more frequently detected in sewage than some other bacteria defoliated from human skin, such as methicillin-resistant *Staphylococcus aureus* (MRSA) and the opportunistic pathogens *P. aeruginosa* and *A. baumannii.* Also, if a pathogen load per infected individual is low, a high number of infected individuals may be needed to detect the pathogen in a sewerage system. Further, an ‘outbreak’ of a resistant pathogen can be as few as a single patient, and in that case, it is difficult to detect with WWS.

The load of ARB from environmental sources, such as biofilms growing in sewage infrastructure, can contribute to ARB detected from wastewater. One earlier study demonstrated that not all carbapenemase-producing *Enterobacteriaceae* in hospital wastewater originated from a human source, but instead, some of the strains could originate from biofilms from within the sewage system such as pipes (White et al., 2016). Manageiro et al. detected GES-5-producing *K. pneumoniae* in water systems and suspected aquatic environments as a potential reservoir for these types of genes (Manageiro et al., 2014). Other studies also suspected the possible environmental origin for carbapenemase genes in a WW environment, as the presence of GES and NDM-producing *Enterobacteriaceae* in wastewater did not correspond with clinical cases (White et al., 2016; Tiwari et al., 2022e). This means WWS of ARB is challenging to use, regarding bacteria being able to maintain and develop in the sewage system by themselves with existing monitoring technologies. Thus, comparing WWS of ARB to clinical evidence as it has been done for SARS-CoV-2 is not easy. It demands a comprehensive expert interpretation, including an assessment of current events circulating in the community and potential consequences affecting public health.

Furthermore, HGT of mobile genetic elements, particularly plasmids carrying clinically relevant ARG, can find their way into diverse environmental bacteria that are well adapted to survive outside of human and animal hosts and hence can act as an environmental reservoir. Subsequently, such environmental bacteria can transfer these mobile genetic elements harboring ARGs back to the next clinically relevant pathogen and add more complications for relating sewage monitoring to clinical cases. Still, the detection of ARB pathogens in sewage can form part of environmental surveillance dominated by human sources. According to the One Health perspective, ARB from one compartment among humans, animals, or the environment can easily transfer to the other compartments through food, water, and other routes of contamination.

Wastewater surveillance of different bacterial pathogens, irrespective of their resistance profile, has also been investigated. Yan et al. reported a positive relationship between clinical cases of salmonellosis and *Salmonella* counts in wastewater in Honolulu, Hawaii, USA (Yan et al., 2018). The main differences between studies that only targeted ARB isolates and those that targeted all wild types (i.e., without considering the resistance profile) of a bacterial pathogen are that under normal (non-epidemic) conditions, ARB pathogen can be less abundant than wild pathogen types, and ARB pathogen and wild type pathogens may differentially survive in various environmental conditions.

### Monitoring methods

The culture-based approach was more common in the reviewed literature than other methods, especially at the sewershed level and the national and regional levels. This can be because the current AMR surveillance approach is based on clinical isolates, and most of the reviewed studies were conducted to evaluate the human health risks due to the environmental release of AMR via wastewater. Furthermore, adopting a culture-based approach can be relatively more convenient at a local level, as the storing and shipping of large volumes of samples for long distances can be relatively challenging. Comparatively, for molecular methods extracting DNA/RNA, relatively small volumes could be frozen and shipped over long distances to research centers abroad. In addition, the availability of clinical data appears to be a limiting factor for the WWS of ARB validation purposes and enough resources should be ensured in the phase of building-up the WWS of ARB systems to enable correct interpretation of the generated data.

The culture-based approach is a reliable method for providing proof of viable bacteria of concern (Mclain et al., 2016; Schmiege et al., 2021). The use of general media provides an advantage in the susceptibility testing of each isolate (Ferreira da Silva et al., 2007). Furthermore, the selective media supplemented with antibiotics used in many studies provide proof of viable bacteria having reduced susceptibility to certain antibiotics (Watkinson et al., 2007; Schmiege et al., 2021). These approaches yield information on phenotypic isolates as well as their resistance patterns and provide important insights into the genotype-phenotype relationship. Additionally, the combined use of WGS provides high-resolution genotypic profiling and thus allows determining the location of AMR genes inside the bacterial genome and therefore their genetic support (Manageiro et al., 2014). The culturing of an isolate and analysis of its genes may facilitate the detection of new resistance genes or clarify the limited perspective given by the assessment of the environmental resistome. However, culture-based methods have some limitations for consideration. Only less than 1% of environmental microbes can be cultured. A large proportion of gut bacteria are anaerobic, and therefore may rapidly die under ambient environmental conditions, and most of them are difficult to culture. Thus, applying a culture-based method may not capture most of these bacteria in sewage (Firoozeh and Zibaei, 2020). These groups of bacteria can act as a pool for ARB, as HGT within and across *Bacteroidota* and other species has been reported (Shoemaker et al., 2001).

Relatively few studies (only three) have reported PCR-based approaches with qPCR (Bich et al., 2019), high-throughput qPCR (Pärnänen et al., 2019), or dPCR (Mtetwa et al., 2021). These approaches can overcome some limitations of culture-based methods and can detect microbes that are difficult to culture (Tiwari et al., 2022b). Often, these methods are more sensitive, specific, and accurate than culture-based methods for monitoring targets (Sinclair et al., 2008; Tiwari et al., 2021b) because they enumerate viable but not culturable (VBNC) targets, and they can also monitor extracellular DNA in the environment, which is prone to HGT. But if a target is fully culturable, then culture-based methods were reported more sensitive than the qPCR method (Bliem et al., 2018). Also, as a limitation of the PCR-based method, prior information about the target sequence is needed for PCR assay design. However, comparing the pros and cons of different methods is not so simple. All methods have their strengths and limitations.

Other studies have used shotgun metagenomics for WWS of ARB. This approach was mostly used for surveillance over a large geographical area (Forslund et al., 2013; Petersen et al., 2015; Hendriksen et al., 2019b; Riquelme et al., 2021). It yields the bacterial diversity and abundance, and an inventory of all ARGs as a comprehensive overview of the environmental resistome, an abundance of ARG reads that confers resistance to antibiotic classes (Manageiro et al., 2014; Hendriksen et al., 2019c). As the prevalence of different ARGs is not equally distributed and important from a public health perspective for all antibiotic classes. However, such limitations can be overcome by primer-based studies mainly targeting clinically relevant resistance genes. Further, if frozen samples need to be transported for metagenomic analysis, it may impact the target analysis in terms of intracellular and extracellular gene fragments. This is mostly overlooked at present in the existing literature (Dai et al., 2022; Liguori et al., 2022; Thakali et al., 2022).

Whole-genome sequencing has been used to detect ARGs in a specific isolate. Pure culture followed by WGS has been demonstrated to be effective for determining the connection between phenotypic and genotypic profiles. It can provide insights into pathogen transmission and support outbreak investigations based on core-genome phylogenetic analysis but may not enable detailed characterizations of plasmids and their environmental transmission. The use of long-read sequencing platforms such as the Pacific BioSciences RSII system (PacBio) and the Oxford Nanopore Technologies (ONT) MinION system can relatively more capable for determining the exact location of ARG either to the chromosome or plasmids in the host bacteria than traditional shotgun sequencing technologies (Dai et al., 2022; Miłobedzka et al., 2022). All traditional DNA-based methods have the common limitation, that they cannot differentiate between viable and dead cells as DNA can also persist for a certain amount of time in dead cells or even as extracellular DNA (Nocker and Camper, 2006; Zaiko et al., 2018; Rytkönen et al., 2021). Some types of bacterial genome material (for example ribosomal RNA) may reduce their copies in the stressed stage, before their death, but other genome materials (genomic DNA) may persist many days after their death (Brooks and Field, 2016). Dupray et al. reported DNA can persist up to 55 days at 10 °C and 2-10 days at 20 °C after losing the culturability of *Salmonella* (Dupray et al., 1997). In the same way, different ARG can have various decay rates in the ambient wastewater environment.

## Limitations of wastewater surveillance and future direction

Unlike viruses, bacteria can multiply and replicate outside of host cells. Thus, it is difficult to correctly estimate the prevalence of the disease in the population by monitoring ARB in wastewater, especially for gene-based ARG studies, which should be differentiated here from culture-based studies on multi-drug resistant pathogens. ARB pathogen detected in wastewater could have been contributed by animal sources (meat and egg production facilities, agricultural farms, or pets). The detection of ARB and related genes in wastewater cannot indicate the possible source of contamination. However, monitoring ARB together with host-specific primers (Hultman et al., 2018; Diebold et al., 2021), and using microbial source tracking methods may help in predicting the load from non-human sources (Green et al., 2014; Harwood et al., 2014; Rytkönen et al., 2021). Detection of such sources is important for the remediation of contamination and can be helpful for the actual estimation of the prevalence of ARB at a population level.

Furthermore, due to the lack of common guidelines and standard protocols for data collection and analysis, the results obtained from different laboratories are highly sporadic and fragmented, so it is difficult to compare them. As mentioned in earlier sections, the source of ARB pathogens, their fate, and possible effects resulting from changes in sewage flow due to rain, snowmelt, and industrial discharges are poorly understood. Urgently, the development of a standard protocol for WWS of ARB, and suitable normalization procedures (to account for changes in sewage flow and industrial discharges) can make the use of WWS of ARB pathogen easier for sharing and comparing research findings in both vertical timeframes in an area or horizontal geographical spans between different areas.

### More comparison studies

More studies comparing WWS of ARB and clinical cases are needed, considering the occurrence, fate, and decay of each ARB separately in the sewerage system. Further, the estimation of the number of infected individuals needed for detecting a pathogen in a sewage system and how the detection and quantification threshold of such pathogen changes in sewage with an increase in the number of infected individuals in communities can help to interpret WWS of ARB and relate it to clinical cases (Tiwari et al., 2022d). Comprehensive information about how different ARB interacts in wastewater distribution systems can enables the interpretation of WWS of ARB simpler, easier, more straightforward, uniform, and universal. However, accounting for the contribution of ARB and related genes in a community by temporary travelers is challenging.

### Consensus on the interpretation of results and uniformity of monitoring methods

Currently, there is no benchmark or threshold for WWS of ARB regarding how much (diversity and abundance of pathogens) is too much and when to raise a red flag (declare an outbreak or emergency state). Studies comparing wastewater-based findings with clinical evidence may help to generate the correct interpretation of WWS of ARB. However, interpreting the results obtained from molecular methods is challenging. As the copy numbers of different ARGs or even the same ARG can vary in different bacteria. So, establishing a relationship between the CFU of an ARB with its GC of ARG is highly challenging. Such a relationship can be useful for public health risk assessment. Some earlier studies proposed normalizing the ARGs with the 16S rRNA gene for both qPCR and high-throughput shotgun metagenomic sequencing for simplifying the comparison of ARGs in different samples (Graham et al., 2011; Tiwari et al., 2022c). A greater consensus in interpreting wastewater-based findings and uniformity in surveillance methods and the isolation of ARB pathogens at global or regional levels make it easier to share research findings and compare risks.

### Developing antibiotic-resistant bacterial indicators

Developing ARB indicator(s) for surveillance purposes could make the process easier and help to establish uniformity in the way fecal indicator bacteria are used for monitoring the microbial quality of surface waters throughout the world. An ideal indicator would represent a maximum number of ARB pathogens, with positive relationships with them. Such indicators need to be detected more frequently than many ARB pathogens and with a high number during monitoring. However, getting such an ideal indicator is challenging as, each ARB pathogen has a unique source, fate, and decay characteristics in sewerage systems. Many studies have reported that antibiotic-resistant *E. coli* could possess some of these characteristics and could therefore be an option for ARB indicators in the environment and wastewater (Navarro et al., 2014; Kwak et al., 2015; Paulshus et al., 2019; Anjum et al., 2021). Another study proposed that antibiotic-resistant *E. coli, K. pneumoniae, E. faecalis*, and *E. faecium* can be used as indicators of fecal origin, and *Aeromonas* spp. and *P. aeruginosa* as indicators of nosocomial origin (Supplementary Table 3; Berendonk et al., 2015). Several other ARGs have been proposed as indicators for monitoring environmental samples (Supplementary Table 4). Among them, Class 1 integrons are regarded as a marker of sewage impact (Gillings et al., 2015).

### Regular surveillance globally

Many emerging and existing bacterial pathogens need regular and reliable surveillance at the global level to reduce their public health risks. Such routine surveillance may benefit high-population regions as well as resource-limited countries, as it can provide crucial information on ARB circulating in communities. However, still a lot needs to be done for developing rapid, easy, and cost-effective ARB surveillance tools as WWS. Such tools and methods can make WWS more accessible, especially in resource-limited settings. In the same vein, WWS of ARB for both vertical and longitudinal levels of transmission could make the comparison of trends easier and help in identifying ARB hot spots. Furthermore, regular monitoring may help in tracking the possible transboundary movement of the ARB pathogen (Petersen et al., 2015; Ahmed et al., 2020).

### Factors affecting the loading and fate of antibiotic-resistant bacterial and antibiotic resistance genes in sewer networks

Accounting for the factor affecting the loading and fate of ARB and ARG in sewer networks and factors affecting reaching such bacteria and genes from infected individuals to the sewage sample collection points is important for the reliable use of WWS of ARB. Further, knowing the factors affecting the proliferation of ARB in wastewater distribution systems may help public health researchers to understand the dynamics of ARB pathogens and their extracellular DNA harboring ARGs in distribution systems. The adverse environmental conditions meet by certain ARB pathogens in the environment and their subsequent die-off may push them below the radar.

Biological factors particularly bacteriophages have an important role in transferring ARGs in wastewater, not only for limiting the proliferation of host bacteria, but also help for HGT of ARG through transduction (Vrancianu et al., 2020; Maganha de Almeida Kumlien et al., 2021). Further, different biocidal agents such as antibiotics, heavy metals, detergents, and pesticides present in wastewater have a combined effect in creating selective ecological pressure for the proliferation of ARB. One study reported a higher resistance rate of vancomycin-resistant *Enterococci* in wastewater isolates (14.3%) than in community fecal isolates (6.2%) (Haghi et al., 2019). The reduction of ARB pathogen in wastewater effluent after treatment is mostly lower than the reduction of wild types of the same pathogen (Karkman et al., 2018; Miłobedzka et al., 2022). The role of different stress factors in the fate and decay of ARB and related genes needs to be investigated.

Furthermore, many environmental factors, such as temperature, can affect the HGT of ARGs. Conjugative ARG transfer was reported to be more common at 30°C than at 25°C or 37°C (Botes et al., 2013). Another study reported that a high temperature (∼41–45 °C) significantly promotes cell-to-cell plasmid transformation between *E. coli* cells in mixed culture, i.e., biofilms and liquid culture (Hashimoto et al., 2019). At the optimum temperature, the frequency of plasmid transfer is 1,000 times higher than under natural ambient atmospheric conditions (Hashimoto et al., 2019). Calero-Cáceres and Muniesa (2016) conducted a mesocosm study at different temperatures and pH levels and reported that ARB was more strongly affected by temperature than pH (Calero-Cáceres and Muniesa, 2016). The effect of various environmental factors on the HGT of ARGs needs to be investigated more comprehensively. This would allow the detection of ARGs in wastewater to be interpreted more accurately.

### Limitations of the current systematic review

Major limitations of this systematic review were the high heterogeneity among the reviewed studies in terms of sample size, target, and detection methods, as well as the lack of transparency of the methodology. There could be reporting biases (most likely positive and statistically significant results could be more frequently published than negative and non-significant ones), together with target selection bias, inadequate blinding, attrition bias, and publication bias in reviewed earlier studies (Tiwari et al., 2021c). Furthermore, many of the studies that were reviewed comparing WWS with clinical evidence were designed to protect public health by targeting health-related isolates rather than aiming at actual validation of WWS.

In addition, the availability of clinical data appears to be a limiting factor for the WWS of ARB validation purposes. Clinical information at an individual level is a sensitive privacy issue. Hospital clinical reported data can be a source of reliable reference information for comparing with the WWS finding, but as mentioned earlier, hospital data represents only a fraction of the problem. Hospital wastewater is considered the next option, but also includes relatively more ARB from clinical isolates (Perry et al., 2019, 2021; Majlander et al., 2021; Hutinel et al., 2022). In addition, the geographical region of the sewerage network and hospital coverage area may not necessarily overlap (Karkman et al., 2020; Tiwari et al., 2022d). Therefore, harmonization of the WWS of ARB validation and monitoring methodology is challenging, but it is beneficial for a meaningful comparison of the findings at the national and international levels.

## Summary and conclusion

Antibiotic-resistant bacterial surveillance has two major aims: to evaluate the overall ARB burden in a given environment and to evaluate the biological hazards, especially the ARGs of special clinical concern. Tentatively, WWS of ARB fulfills both aims. WWS of ARB has significant advantages over the traditional clinical resistance-based surveillance approach, by providing near real-time information on the ARB situation and has the potential to provide an early prediction of new ARB pathogen threats, which is not the case with the current surveillance approach relying on annual reports of clinical cases. However, WWS of ARB has not yet been fully developed and still needs many improvements before using this approach as a reliable tool for evaluating ARB burden and associated hazards. Urgently, the development of a standard protocol for WWS of ARB, and suitable normalization procedures (to account for changes in sewage flow and industrial discharges) are needed, which could make it easier for sharing and comparing research findings in both vertical timeframes in an area or horizontal geographical spans between different areas. Also, the use of WWS of ARB for regulatory purposes throughout the world may help in coping with the challenge of antibiotic resistance. Indeed high-quality, comprehensive, and real-time surveillance data can help to reduce the burden of ARB (Tacconelli et al., 2018b). WWS of ARB can have the potential to work as a reliable complementary tool for the currently used clinical isolate-based approach. However, the interpretation of WWS of ARB can be not always straightforward; so enough resources should be ensured in the phase of building-up the WWS of ARB systems to enable correct interpretation of the generated data.

Different methods are available for WWS of ARB, but each of them has its own advantages and disadvantages, and no single method can fulfill the task on its own. The integrated use of all qPCR, culture-based and metagenomic methods can provide holistic information in ARB surveillance (Cai et al., 2021). Culture-based methods are the most frequently used for comparing WWS of ARB with clinical evidence. The metagenomic surveillance approach provides resistome information covering the whole community, so it is difficult to state which genetic marker correlates best with clinical resistance to the different classes of antibiotics in infections caused by a given pathogen. Thus, sewage metagenomic data cannot be an alternative to clinical surveillance, as the current metagenomics methods cannot directly link resistance genes to the bacterial hosts (Karkman et al., 2020). However, molecular methods are continuously advancing over time, and it can be possible in the future. For example, the use of long-read sequencing (PacBio & MinION) can indicate the source bacteria of ARG (Qian et al., 2021; Dai et al., 2022). Determining the performance characteristics of WWS of ARB targets, such as target sensitivity and specificity can make it simpler for interpreting results. Therefore, further studies for evaluating its performances, considering the occurrence, fate, and decay of each ARB pathogen separately in the sewage system can be beneficial for the advancement of WWS of ARB. Such studies may also help in interpreting the WWS of ARB and in understanding the strengths and limitations of the approach. Also, future studies evaluating the occurrence of each ARB pathogen in the sewerage system and comparing results to the clinical infections caused by these pathogens can make it possible to evaluate the usability and power of WWS in the surveillance of ARB pathogens circulating in a community.

## WastPan study group

WastPan consortium members are entitled for authorship: Annika Länsivaara, Rafiqul Hyder, and Erja Janhonen from Faculty of Medicine and Health Technology, Tampere University, Tampere, Finland; Anna-Maria Hokajärvi, Anniina Sarekoski, Aleksi Kolehmainen from Expert Microbiology Unit, Finnish Institute for Health and Welfare, Kuopio, Finland; Soile Blomqvist, Kati Räisänen and Carita Savolainen Kopra from Expert Microbiology Unit, Finnish Institute for Health and Welfare, Helsinki, Finland; Teemu Möttönen, Oskari Luomala, Aapo Juutinen from Infectious Disease Control and Vaccinations Unit, Finnish Institute for Health and Welfare, Helsinki, Finland. Ocean Thakali, Shyam Kumar Mishra are invited authors and are not belong WastPan consortium.

## Author contributions

AT conceptualized and designed the study, analyzed the data, and drafted the initial version of the manuscript. PK, VH, AA-M, and VJ contributed to the introductory section. AA-M, OT, and SM brought an international point-of-view on the interpretation of the systematic review outcomes. SO, K-ML, TP, and AH acquired funding and supervised the project and allocated the resources. The WastPan Study Group devoted to developing wastewater-based surveillance as a pandemic preparedness tool provided the wider research environment for the work. All authors reviewed the manuscript and approved the final submitted version. All authors approved the submitted version.
