# Supplementary material for: Wastewater surveillance of antibiotic-resistant bacterial pathogens: A systematic review
Source: Front Microbiol. 2022 Dec 15;13:977106. doi: 10.3389/fmicb.2022.977106 (PMC9798455; doi:10.3389/fmicb.2022.977106)
Supplement: Supplementary file 1 [file Data_Sheet_1.pdf]

Supplemental File

Wastewater Surveillance of Antibiotic-Resistant bacterial pathogens: a Systematic Review

Ananda Tiwari[1*], Paula Kurittu[1], Ahmad I. Al-Mustapha[1,2,3], Viivi Heljanko[1], Venla Johansson[1], Ocean Thakali[4], Shyam Kumar Mishra[5], Kirsi-Maarit Lehto[6], Anssi Lipponen[7], Sami Oikarinen[6], Tarja Pitkänen[1,7], WastPan Study Group, Annamari Heikinheimo[1,8*]

[1]Department of Food Hygiene and Environmental Health, Faculty of Veterinary Medicine, University of Helsinki, Helsinki, Finland.

[2]Department of Veterinary Public Health and Preventive Medicine, Faculty of Veterinary Medicine, University of Ibadan, Oyo State, Nigeria.

[3]Department of Veterinary Services, Kwara State Ministry of Agriculture and Rural Development, Ilorin, Kwara State, Nigeria.

[4]University of Ottawa, Department of Civil Engineering, Ottawa, Canada.

[5]School of Optometry and Vision Science, University of New South Wales Sydney, Australia.

[6]Faculty of Medicine and Health Technology, Tampere University, Tampere, Finland.

[7]Department of Health Security, Expert Microbiology Unit, Finnish Institute for Health and Welfare, Finland.

[8]Finnish Food Authority, Seinäjoki, Finland.

[In addition to the authors listed above, the following WastPan consortium members are entitled for authorship: Annika Länsivaara, Rafiqul Hyder, and Erja Janhonen from Faculty of Medicine and Health Technology, Tampere University, Tampere, Finland; Anna-Maria Hokajärvi, Anniina Sarekoski, Aleksi Kolehmainen from Expert Microbiology Unit, Finnish Institute for Health and Welfare, Kuopio, Finland; Soile Blomqvist, Kati Räisänen and Carita Savolainen Kopra from Expert Microbiology Unit, Finnish Institute for Health and Welfare, Helsinki, Finland; Teemu Möttönen, Oskari Luomala, Aapo Juutinen from Infectious Disease Control and Vaccinations Unit, Finnish Institute for Health and Welfare, Helsinki, Finland. Ocean Thakali, Shyam Kumar Mishra are invited authors and are not belong WastPan consortium].

Supplementary Material 1 (SM1)

Literature search was made with the following eleven search keywords pairs:

a. [“Wastewater” OR “Sewage”] and [“Antimicrobial Resistance” OR “Antibiotic-Resistant” OR “Antimicrobial-resistant” or “Antibiotic Resistance”]
b. [“Wastewater” OR “Sewage”] and [“Carbapenemase” OR “Carbapenem-Resistant” or “Carbapenem Resistance”]
c. [“Wastewater” OR “Sewage” and “Beta-lactamase” OR “Beta-lactam” or “ESBL”]
d. [“Wastewater” OR “Sewage”] and [“Amoxicillin Resistance” OR “Amoxicillin Resistant”]
e. [“Wastewater” OR “Sewage”] and [“Aminoglycoside Resistance” OR “Aminoglycoside Resistant”]
f. [“Wastewater” OR “Sewage” and “Cephalosporins Resistance” OR “Cephalosporins Resistant”]
g. [“Wastewater” OR “Sewage” and “Ertapenem Resistance” OR “Ertapenem Resistant”]
h. [“wastewater” OR “Sewage” and “Fluoroquinolones Resistance” OR “Fluoroquinolones Resistant”]
i. [“Wastewater” OR “Sewage” and “Vancomycin Resistance” OR “Vancomycin-Resistant”]
j. [“Wastewater” OR “Sewage” and “Erythromycin Resistance” OR “Erythromycin Resistant”]
k. [“Wastewater” OR “Sewage”] and [“Methicillin Resistance” OR “Methicillin-Resistant”].

Supplementary Material 2 (SM2): Flow chart of the phases in the systematic literature review.

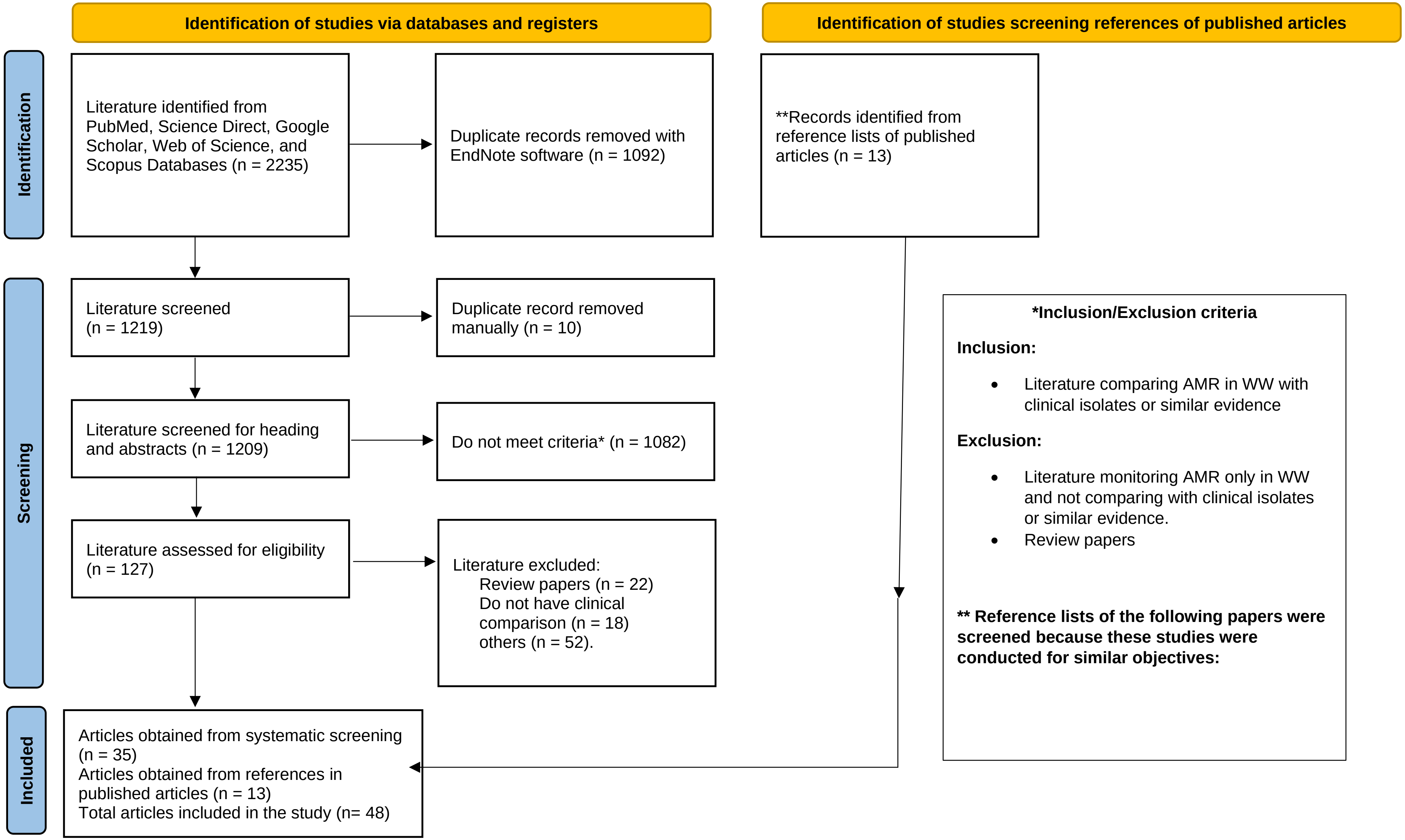

Table S1. Reviewed literature and their geographical coverage, and surveillance methods, and AMR targets.

	International/ intercontinental	International/ continental	National/regional (many cities)	Single sewershed	Hospital	Total
Metagenomics	1. (Auguet et al., 2021),<br>2. (Forslund et al., 2013),<br>3. (Hendriksen, et al., 2019a),<br>4. (Karkman et al., 2020),<br>5. (Petersen et al., 2015)<br>6. (Riquelme et al., 2021),	-	1. (Su et al., 2017)	1. (Majeed et al., 2021)<br>2. (Hendriksen et al., 2019b)	1. (Cai et al., 2021),<br>2. (Perry et al., 2021)	(n = 11)
PCR	-	1. (Pärnänen et al., 2019)	-	1. (Bich et al., 2019),<br>2. (Mtetwa et al., 2021)	-	(n = 3)
Culture	-	1. (Huijbers et al., 2020)	1. (Blaak et al., 2021),<br>2. (Gouliouris et al., 2019),<br>3. (Meir-Gruber et al., 2016)<br>4. (Moradigaravand et al., 2018),<br>5. (Reinthaler et al., 2013),<br>6. (Urase et al., 2020)	1. (Abduljabbar & Aljanaby, 2018)<br>2. (Adator et al., 2020)<br>3. (Diemert & Yan, 2019),<br>4. (Golle et al., 2017),<br>5. (Haghi et al., 2019)<br>6. (Hutinel et al., 2019),<br>7. (Jakobsen et al., 2008),<br>8. (Jørgensen et al., 2017)<br>9. (Khan et al., 2019)<br>10. (Kolokotsa & Leotsinidis, 2020),<br>11. (Mourkas et al., 2019),<br>12. (Ojer-usoz et al., 2017),<br>13. (Oravcova et al., 2017)<br>14. (Paulshus et al., 2019),<br>15. (Pignato et al., 2010),<br>16. (Rahimi & Bouzari, 2015),<br>17. (Raven et al., 2019),<br>18. (Rodríguez et al., 2021),<br>19. (Saifi et al., 2009),<br>20. (Talebi et al., 2008),<br>21. (Tiwari et al., 2022),<br>22. (Yang et al., 2009),<br>23. (Zaheer et al., 2020),<br>24. (Zarfel et al., 2013)	1. (Drieux et al., 2016),<br>2. (Flach et al., 2021),<br>3. (Yang et al., 2009)	(n = 34)
Escherichia coli	-	1. (Huijbers et al., 2020)	1. (Reinthaler et al., 2013)	1. (Adator et al., 2020)[Ω]<br>2. (Hutinel et al., 2019),<br>3. (Jakobsen et al., 2008),<br>4. (Jørgensen et al., 2017),<br>5. (Kolokotsa & Leotsinidis, 2020)[#],<br>6. (Ojer-usoz et al., 2017),<br>7. (Paulshus et al., 2019),<br>8. (Pignato et al., 2010)*<br>9. (Raven et al., 2019),<br>10. (Urase et al., 2020)[ΩΩ],	-	(n = 14)

				11. (Yang et al., 2009), 12. (Zarfel et al., 2013),		
Vancomycin-resistant *Enterococcus* spp.	-	-	1. (Gouliouris et al., 2019)	1. (Haghi et al., 2019), 2. (Kolokotsa & Leotsinidis, 2020)[#], 3. (Oravcova et al., 2017), 4. (Saifi et al., 2009), 5. (Talebi et al., 2008), 6. (Zaheer et al., 2020)	-	(n = 7)
CRE	-	-	1. (Blaak et al., 2021), 2. (Meir-Gruber et al., 2016)	1. (Adator et al., 2020)[Ω] 2. (Tiwari et al., 2022), 3. (Urase et al., 2020)[ΩΩ]	1. (Flach et al., 2021)	(n = 6)
ESBL	-	-	1. (Urase et al., 2020)	1. (Rodríguez et al., 2021)	1. (Drieux et al., 2016)	(n = 3)
Pseudomonas spp.	-	-	-	1. (Abduljabbar & Aljanaby, 2018) 2. (Golle et al., 2017), 3. (Kolokotsa & Leotsinidis, 2020)[#],	-	(n = 3)
Staphylococcus aureus	-	-	1. (Meir-Gruber et al., 2016)	1. (Kolokotsa & Leotsinidis, 2020)[#], 2. (Rahimi & Bouzari, 2015)	-	(n = 3)
Salmonella spp.	-	-	-	1. (Diemert & Yan, 2019) 2. (Pignato et al., 2010)*,	-	(n = 2)
*Clo*stridium difficil*e*	-	-	1. (Moradigaravand et al., 2018)	-	-	(n = 1)
Coliforms and other Gram-negative bacteria	-	-	-	1. (Khan et al., 2019)	-	(n = 1)
Campylobacter spp.	-	-	-	1. (Mourkas et al., 2019)	-	(n = 1)

Studies with similar [Ω, #, ΩΩ, *] were conducted for more than one targets.

Table S2. Reviewed literature and clinical studies used for comparing against wastewater-based studies.

**Independent clinical data from Hospital**	**Antibiotic consumption at Global level is considered**	**Antibiotic consumption on daily basis is followed**	**ERAS-Net**	**Hospital area vs without hospital area**	**Government**	**Previously published research**
(Auguet et al., 2021)<br>(Cai et al., 2021)<br>(Drieux et al., 2016)<br>(Flach et al., 2021)<br>(Golle et al., 2017)<br>(Gouliouris et al., 2019)<br>(Haghi et al., 2019)<br>(Hutinel et al., 2019)<br>(Jørgensen et al., 2017)<br>(Majeed et al., 2021)<br>(Moradigaravand et al., 2018)<br>(Mourkas et al., 2019)<br>(Ojer-usoz et al., 2017)<br>(Oravcova et al., 2017)<br>(Perry et al., 2021)<br>(Rahimi & Bouzari, 2015)<br>(Raven et al., 2019)<br>(Reinthaler et al., 2013)<br>(Saifi et al., 2009)<br>(Talebi et al., 2008)<br>(Yang et al., 2009)<br>(Zaheer et al., 2020)<br>(Zarfel et al., 2013)	(R. S. Hendriksen et al., 2019)<br>(Pärnänen et al., 2019)	(Bich et al., 2019)<br>(Mtetwa et al., 2021)<br>(Su et al., 2017)	(Huijbers et al., 2020)<br>(Karkman et al., 2020)<br>(Kolokotsa & Leotsinidis, 2020)<br>(Pärnänen et al., 2019)<br>(Tiwari et al., 2022)	(Blaak et al., 2021)<br>(Khan et al., 2019)	(Meir-Gruber et al., 2016)<br>(Urase et al., 2020)	(Rodríguez et al., 2021)<br>(Tiwari et al., 2022)

Table S3. AMR indicators (Berendonk et al., 2015)

**Species**	**Taxonomic division**	**Primary habitat**	**Indication**
Escherichia coli	*Gammaproteobacteria* (C)_ *Enterobacterales* (O)_ *Enterobacteriaceae*(F)	Gut of warm-blooded animals, opportunistic pathogen	Fecal origin
Klebsiella pneumoniae	*Gammaproteobacteria* (C)_ *Enterobacterales* (O)_ *Enterobacteriaceae*(F)	Gut of warm-blooded animals, opportunistic pathogen	Fecal origin
Aeromonas spp.	*Gammaproteobacteria* (C)_ Aeromonadales (O)_ Aeromonadaceae (F)	Environmental	Nosocomial
Pseudomonas aeruginosa	Gammaproteobacteria (C)_ Pseudomonadales (O)_ Pseudomonadaceae (F)	Environmental and a common skin flora	Nosocomial
Enterococcus faecalis	Bacilli(C)_ Lactobacillales (O)_Enterococcaceae (F)	Gut of warm-blooded animals, opportunistic pathogen	Fecal origin
Enterococcus faecium	Bacilli(C)_ Lactobacillales (O)_Enterococcaceae (F)	Gut of warm-blooded animals, opportunistic pathogen	Fecal origin

C = Class, O = Order, F= Family

Table S4. ARG indicators of AMR (Berendonk et al., 2015)

**Genes**	**Characters and explanation**
*intl*1	Integrase gene of class 1 integrons, a genetic platform for ARG capture
*sul*1 & *sul*2	Sulfonamide-resistant dihydropteroate synthase
$bla_{CTX\text{-}M}$ & bla_{TEM}	β-lactamases, frequently identified in *Enterobacteriaceae*
$bla_{NDM\text{-}1}$	New Delhi metallo-β-lactamase
bla_{VIM}	Carbapenemase, frequent in clinical *Pseudomonas aeruginosa* in certain areas
bla_{KPC}	*Klebsiella pneumoniae* carbapenemase
*qnr*S	Quinolone pentapeptide repeat family
aac-(6′)-Ib-cr	*Aminoglycoside acetyltransferase*
*van*A	Vancomycin resistance operon gene
*mec*A	Penicillin binding protein
*erm*B & *erm*F	rRNA adenine N-6-methyltransferase, associated with macrolide resistance
*tet*M	Ribosomal protection protein, associated with tetracycline resistance
aph	Aminoglycoside phosphotransferase

Table S5. Wastewater surveillance studies with inferences regarding the population or drug consumption.

Sample included	ARB target	Method	Major results	Reference
**International (many countries)**				
Pooled fecal samples (Kenya, n = 177; UK, n = 157; Cambodia, n = 156); independent clinical data (Kenya, n= 910; UK, n = 3356; Cambodia, n = 197).	ARG Metagenome	● Metagenomic analysis, compared *Enterobacterales* bacterial family, order, and species from individual stool samples and compared the prevalence of these microbes in blood and cerebrospinal fluid samples.	● The abundance of targeted bacteria varied in the samples collected from different countries.<br>● Kenya had lower numbers of enterobacteria in fecal samples, but numbers were similar in clinical isolates from all three countries.<br>● Klebsiella reads were higher in Cambodia than in the UK and Kenya in both fecal and clinical samples.<br>● In cerebrospinal fluid samples, *Salmonella* spp. was higher in Kenya than in Cambodia and the UK.	(Auguet et al., 2021)
Stool samples collected in Denmark, Spain, & USA from earlier projects were used.	ARG Metagenome	● 71 Danish and 39 Spanish samples, 139 U.S. samples.<br>● Metagenomics was used for analysis.	● 68 classes and subclasses of antibiotic resistance genes were detected.<br>● The most abundant resistance was related to antibiotics that were used in both humans and animals and that had been available for a long time.	(Forslund et al., 2013)
Resistomes of sewage influent from 79 sites in 60 countries and six continents.	ARG Metagenome	● 24-h flow proportional sampling or composites (3 sub-samples) over a short period (minimum 15 min).<br>● World Bank Health, Nutrition, Population, Development indicator data sets were compared with resistomes.<br>● Metagenome was compared with antibiotic use, and socio-economic, health, and environmental factors.	● Antibiotic use and bacterial taxonomy only explained a minor part of the variation in the resistome.<br>● Global resistome diversity and abundance varied by region.<br>● Resistome abundance strongly correlated with country-specific variables from the World Bank's Health, Nutrition and Population, Development indicator data sets (mostly sanitation and general health).<br>● Countries with a higher HDI have a significantly lower abundance of ARG, and the number of passengers flying into a country does not affect this abundance.<br>● No significant association was detected between temperature at collection and ARG abundance, or between the abundance of ARG on a class level and the antibiotics residue levels measured.	(R. S. Hendriksen et al., 2019)
79 sites in 60 countries and six continents.	ARG Metagenome	● Metagenomic analysis (used the same sequence data produced by [65]<br>● They downloaded the raw sequence data from a database and compared the sewage metagenomic data with clinical ARB prevalence using environmental and clinical surveillance data on invasive *E. coli* isolates.<br>● Clinical resistance data for *E. coli* (blood, CSF) against four classes of antibiotics extracted from networks EARS-Net, CAESAR and ResistanceMap.	● Established the potential for WW metagenomic data and environmental data in assessing the prevalence of clinical ARB.<br>● WW resistome correlated to clinical surveillance data on invasive *E. coli* isolates, but the relationship was weak.<br>● Among the compared ARGs, the relative abundance of *intI1* performed better than other ARGs.<br>● Data related to the wastewater-based metagenome had a lower model fit than socioeconomic factors.	(Karkman et al., 2020)

Pooled airplane-toilet samples from 18 flights arriving in Copenhagen from Bangkok, Beijing, Islamabad, Newark, Greenland, Tokyo, Toronto, and Washington DC; representing Asia and North America.	ARG Metagenome, bacterial pathogens and noroviruses	● Bacterial DNA and RNA were extracted.<br>● For bacterial DNA, metagenomics was carried out and<br>● for viral RNA, RT-PCR was conducted.	● Asian samples had a higher abundance and richness of ARGs than those from North America.<br>● A total of 31 ARGs were found to significantly differ between geographical areas.<br>● South Asian samples had higher $bla_{\text{CTX-M}}$, *qnrS* and the 16 S methyltransferase *npmA* than those from North America.<br>● Unique reads mapping to the human pathogens *S. enterica*, *C. difficile*, and *C. jejuni* were observed in all samples.<br>● *C. jejuni* was distributed in all areas, but *S. enterica* was more prevalent in South Asia than North Asia and North America.<br>● *C. difficile* was more prevalent in North America and North Asia than South Asia.	(Petersen et al., 2015)
14 WW samples were collected from six countries (from Asia, Europe, & North America).	ARG Metagenome	● Metagenomics and measured antibiotic concentration in WW.	● Variation in bacterial communities and ARG profiles was observed between EU/US vs Asian countries.<br>● $bla_{\text{OXA-type}}$ was ubiquitous and had a relatively high abundance.<br>● bla_{GES}, bla_{SHV}, $bla_{\text{CTX-M}}$, & bla_{TEM} were ubiquitous but with lower abundances.<br>● The qnrS gene was more prevalent in Asia than in Europe and the US.	(Riquelme et al., 2021)
80 samples were analyzed and a total of 1245 *E. coli* isolates were monitored from 10 European cities.	*E. coli*	● *E. coli* was isolated from wastewater and susceptibility was tested as done by EUCAST ECDC, and correlation was tested with EARS-Net reported cases.<br>● Resistance against aminopenicillins, fluoroquinolones, third-generation cephalosporins, & aminoglycosides in *E. coli* was tested.	● WW and clinical resistance had strong relationships.<br>● The prevalence of antibiotic resistance was lower in WW than in clinical *E. coli*.<br>● WW monitoring could be used to predict clinical cases.	(Huijbers et al., 2020)
12 WWTPs in seven European countries.	Highly parallel qPCR	● Highly parallel qPCR targeted 229 resistance genes and 25 mobile genetic elements. EARS-Net reported cases are considered as references.	● WW and clinical resistance had strong relationships.<br>● ARB in WW was not correlated with coliform counts or enterococci counts.<br>● WW monitoring could be used to predict clinical cases.	(Pärnänen et al., 2019)
**National or regional level**				
100 WWTPs in the Netherlands, 40% of the Dutch population (n = 341).	Carbapenemase-producing *Enterobacterales*	● Membrane filtration<br>● ChromID CARBA agar & ChromID OXA-48 agar<br>● *E. coli* was enumerated in Tryptone Bile X-glucuronide and ChromID ESBL.<br>● A total of 1162 CRE isolates were analyzed.<br>● Multiplex (PCRs) targeted 9-CARBA-gene types.<br>● They incubated plates at 44 ºC to inhibit the growth of environmental isolates.	● CPE was detected at 89% of the WWTPs, in 87 influents and 53 effluents.<br>● Overall, 15 CPE types were detected.<br>● *E. coli* carrying *blaOXA-48*-like genes were most frequently detected in sewage, while *K. pneumoniae* was most frequently reported in clinical isolates.<br>● *BlaOXA-48*-like-positive-*K. pneumoniae, blaNDM*- or *blaKPC*-positive *E. coli*, and *blaNDM*- or *blaKPC*-positive *K. pneumoniae* were detected.<br>● The wastewater treatment process reduced CPE by 99%.<br>● CPE concentration was positively correlated with WWTP size and the *E. coli* count.	(Blaak et al., 2021)
20 WWTPs in the east of England.	Vancomycin-resistant *Enterococcus faecium* (VREfm)	● Compared the genomes of 423 isolates from wastewater with 187 isolates associated with bloodstream infection at five hospitals in England.	● Widespread distribution of hospital-adapted VREfm beyond acute healthcare settings with an extensive release of VREfm into the environment.	(Gouliouris et al., 2019)
Wastewater samples from four WWTPs in Israel.	Pan-resistant bacteria (*Enterobacteriaceae*, *Staphylococcus aureus*)	● ARB isolates screened from WW with CHROMagarKPC, CHROMagarMRSA, CHROMagarVRE and compared with clinical isolates reported by the government.	● ARB and ARG distribution in WW was similar to the distribution of clinical isolates.<br>● Multiple pan-resistant bacteria, including Carbapenem-resistant *Enterobacteriaceae* carrying bla_{KPC} and $bla_{\text{NDM-1}}$, MRSA and VRE. bla_{KPC} carrying *Klebsiella pneumonia* and *Enterobacter cloacae* were found, but ARB *Enterobacteriaceae* were predominant.<br>● *K. pneumonia*, *Enterobacter* spp., *E. coli*, and *Citrobacter* spp. were the four main CRE isolated from sewage and in clinical samples.	(Meir-Gruber et al., 2016)

			● WW and clinical samples had similar positive rates for *bla*$_{KPC}$ & *bla*$_{NDM-1}$.<br>● VRE was more abundant in sewage than MRSA.<br>● MRSA and VRE were more often present in clinical than in WW samples.	
WW effluent from 18 WWTPs in the UK.	*Clostridioides difficile* and related ARG	● Membrane filtration was used and 186 isolates were analyzed. WGS was used to compare isolates from WW with clinical sequences obtained from the same site	● Multilocus sequence types (STs) identified 19 distinct STs in the clinical collection and 38 STs in the WW collection, with 13 of 44 STs common to both clinical and WW collections. Furthermore, five pairs of common sequence types were obtained from WW and clinical samples.	(Moradigaravand et al., 2018)
WW activated sludge samples (n = 11) from many WWTPs in Austria. Human samples were from urine, sputum, stool, wounds, skin, & respiratory tract.	*E. coli*	● *E. coli* isolated from WW with Chromocult Agar and fecal samples with Endo Agar, then 356 isolates were tested against 15 antibiotics (amoxicillin, amoxicillin/clavulanic acid, piperacillin /tazobactam, cefalotin, cefuroxime-axetil, cefoxitin, cefotaxime, ceftazidime, meropenem, gentamicin, amikacin, trimethoprim-sulfamethoxazole, nitrofurantoin, norfloxacin, and ciprofloxacin). Resistance was also tested with Vitek 2.	● Resistance against trimethoprim-sulfamethoxazole and the two quinolones, ciprofloxacin and norfloxacin, was only found in human samples and not in WW samples.<br>● The WW-based ARB pattern followed the national increment pattern, and gradually increased from 2000 to 2009.	(Reinthaler et al., 2013)
Samples were collected from 12 WWTPs in Tokyo, Japan.	ESBL-producing *E. coli* & carbapenem-resistant *Enterobacteriaceae*	● 17 wastewater samples, 605 *E. coli* isolates tested against CTX, 976 *Enterobacteriaceae* tested against MEPM.<br>● WW-isolated CTX-R *E. coli* and MPEM-R *Enterobacteriaceae* were compared with national surveillance data (both inpatients and outpatients).<br>● Effluent "after chlorination" from 12 WWTPs; nine receive hospital effluent and use activated sludge treatment, two use oxidation ditches in rural areas, and one is a community plant with no medical facilities.<br>● Cultured with selective chromogenic media & identified with 16S rRNA sequencing, disk-diffusion AST.	● The proportion of cefotaxime-resistant *E. coli* was 5.7%, & ESBL-producing *E. coli* comprised 5.3% of the total *E. coli* (chlorinated effluents).<br>● The proportion of CRE was 0.007% with the constituting species of *Klebsiella* spp. and *Enterobacter* spp.<br>● The prevalence of resistance was lower in effluent wastewater isolates (5.7%) compared to clinical surveillance data for both in- (27.5%) and outpatients (17%).	(Urase et al., 2020)
116 WW samples were collected from 32 WWTPs in 17 Chinese cities.	ARG Metagenome	● Metagenomic analysis was conducted to determine ARB variation in different seasons (summer vs winter), economic development levels, and urban geography.	● Winter samples had a higher abundance of bacteria and ARGs.<br>● Summer samples had a higher relative abundance of ARGs than winter samples.<br>● ARG abundance varied as a function of climatic zonation and population density.	(Su et al., 2017)
**Single sewershed level**				
Compared clinical & sewage isolates in Iraq.	*Pseudomonas aeruginosa*	● A total of 120 isolates were collected (60 from sputum of patients & 60 from WW).<br>● Antibiotic susceptibility was tested using the Kirby-Bauer method.<br>● Six virulence ARGs monitored with PCR.	● All isolates were resistant to amoxicillin but susceptible to imipenem.<br>● There was a positive correlation between a high prevalence of virulence-associated genes & an increase in ARB in *P. aeruginosa* isolates.<br>● The prevalence of MDR was significantly higher in clinical isolates than in WW isolates.<br>● Overall, the prevalence of individual antibiotic resistance was higher in clinical isolates.<br>● A non-significant difference was recorded in the prevalence of ESBLs between the two compartments.<br>● 55 clinical (91.6%) & 41 WW isolates (68.3%) were multi-drug resistant.<br>● *PapA*, *FimH*, *feoB*, *iutA*, *hly*, & *kpsMTII* genes were detected in 91.6, 100, 80, 95, 20 and 100% of clinical isolates, respectively, but in WW isolates, the respective figures were 51.6, 68.3, 41.6, 35, 3.3, 5 and 41.6%.	(Abduljabbar & Aljanaby, 2018)

One Health continuum of the beef production system in Alberta, Canada.	Extended-spectrum cephalosporin-resistant (ESCR) & generic *E. coli*	● A total of 705 ESCR and 663 generic *E. coli* isolates were obtained from cattle feces (CFeces), catch basins (CBasins), surrounding streams, beef processing plants, municipal sewage (MSewage), & human clinical specimens (CHumans).<br>● All isolates were tested for antibiotic susceptibility to 9 antibiotics and two clavulanic acid combinations (ampicillin, amoxicillin/clavulanic, ceftiofur, ceftazidime, ceftazidime/clavulanic acid, streptomycin, neomycin, oxytetracycline, florfenicol, trimethoprim/ sulfamethoxazole & sulfisoxazole) using the Kirby–Bauer disk diffusion susceptibility method with CLSI guidelines.<br>● A simplex and two multiplex PCR assays were used to test against *bla*$_{TEM}$, *bla*$_{SHV}$, *bla*$_{OXA}$, & *bla*$_{CMY}$, & *bla*$_{CTX-M}$.	● Human clinical isolates & WW isolates had a similar prevalence of resistance.<br>● ARB prevalence differed among ESCR from various sampling sources (CFeces, CBasins, MSewage, BProcessing, MSewage, and CHumans).<br>● Resistance was higher for ESCR than for generic *E. coli*.<br>● ESCR from CFeces had higher ARB to the tested antibiotics than other sample types.<br>● Both ESCR and generic *E. coli* had a high resistance to certain antibiotics.<br>● Genotypic analyses revealed a similar outcome, with genotype–phenotype agreement ranging from 93.2–100%, depending on the antibiotic.<br>● In both populations, resistance to oxytetracycline and ampicillin was prevalent, while neomycin resistance was the least common.	(Adator et al., 2020)
WW samples (n = 52) from Honolulu, Hawaii, USA.	*Salmonella enterica*	● WGS was conducted for 21 WW isolates and compared with Salmonellosis clinic isolates.	● The genomic characteristics of WW isolates resembled salmonellosis clinical isolates.<br>● WWS worked as an early warning tool.	(Diemert & Yan, 2019)
Two WWTPs and two hospitals samples from Slovenia.	Carbapenem-resistant *Pseudomonas aeruginosa*	● Monthly effluent samples were collected from two WWTPs over a year. Isolation (n = 83) with filtration and culturing.<br>● Suspected colonies were sub-cultured and confirmed with MALDI-TOF.<br>● Susceptibility of such confirmed isolates was tested against ceftazidime, cefepime, piperacillin/ tazobactam, imipenem, meropenem, ciprofloxacin, tobramycin, gentamycin, amikacin, & netilmicin with disc diffusion following the EUCAST procedure.<br>● Clinical isolates (130 from 109 patients) were obtained from respiratory & UTI patients during routine diagnosis.	● For representatives of all 65 pulsotypes, 49 different MLSTs were determined.<br>● Clinical isolates (n = 130) had 38 & WW isolates (n = 83) had 31 pulsotypes.<br>● Only 9 pulsotypes were shared between clinical and WW settings.<br>● Based on MLST types, clinical & WW isolates differed in ARB .<br>● Clinical isolates had the highest resistance to piperacillin/tazobactam (52.3%) and ceftazidime (42.3%), but WW isolates had the highest resistance to ceftazidime (37.1%) and ciprofloxacin (35.5%).<br>● Most isolates were only resistant to imipenem and/or meropenem.<br>● Carbapenem-resistant *P. aeruginosa* was ubiquitous in WWTP effluents and had a higher diversity than clinical samples.	(Golle et al., 2017)
Prevalence in sewage & fecal samples of healthy carriers was compared (Iran).	Vancomycin-resistant *Enterococci* (VRE)	● Fecal specimens of healthy carriers (n = 100) & MWW samples (n = 100) were filtered to isolate enterococci (Trypticase Soy Agar) & susceptibility testing was conducted with CLSI guidelines using the agar dilution method.	● A high frequency of VRE was recorded in municipal sewage (14.3%) than in healthy carriers (6.2%).	(Haghi et al., 2019)
Eight hospital WW (HWW) samples and six municipal WW (MWW) samples were monitored for one year in Gothenburg (Sweden).	*E. coli*	● *E. coli* was isolated from the hospital (n = 721 isolates) and MWW samples (n = 531 isolates).<br>● An inexpensive broth screening method was validated against disk diffusion and applied to determine resistance against 11 antibiotics in sewage isolates.<br>● Resistance data on *E. coli* isolates from clinical samples from corresponding local hospitals and primary care patients were compared with WW-based resistance using linear regression.	● *E. coli* resistance rates derived from HWW and hospital patients strongly correlated (r^2 = 0.95 for urine and 0.89 for blood samples), as did resistance rates in *E. coli* from MWW and primary care urine samples (r^2 = 0.82).<br>● Resistance rates in HWW isolates were close to those in hospital clinical isolates, while resistance rates in MWW isolates were about half of those measured in primary care isolates.<br>● Resistance rates in MWW isolates were more stable between sampling occasions than those from HWW.<br>● WWS can be a low-cost method for complementing current monitoring systems and providing clinically relevant AR data for countries and regions where surveillance is lacking.	(Hutinel et al., 2019)
Hospital and community WWTP in Denmark.	Gentamicin-resistant *E. coli*	● WW and clinical patient samples were collected, *E. coli* was isolated, and ARB was tested with cell isolation and susceptibility testing.	● ARB profiles from hospital patients & HWW sample bacteria were similar.<br>● ARB profiles in patients and HWW were higher than MWW.<br>● Demonstrated that the *aac(3)-II* gene transferred from patient isolates to WW isolates.	(Jakobsen et al., 2008)

Exploration of whether recreational water is a possible source of ESBL-EC & infection in humans. Compared ESBL-EC from urine, recreational water, & WW in Oslo, Norway.	ESBL<br>*E. coli*	● Clinical (n = 94), surface water (n = 167), & WW isolates (n = 91) were studied, & also WGS (n = 267)<br>● Recreational water (inland & coastal) from 4 beaches & WW samples from a nearby WWTP were filtered and cultured on differential & ESBL-selective media.<br>● Antibiotic susceptibility was tested & multi-locus variable number of tandem repeats assay. Selected ESBL-EC strains from recreational water were characterized by WGS & compared with WW & human urine isolates.<br>● The filters were subsequently grown on Brilliance agar & ChromeID ESBL plates.<br>● Purple colonies on Brilliance plates were considered as presumptive *E. coli* CFUs and purple colonies on ChromeID ESBL plates as presumptive ESBL-EC isolates.	● ESBL-EC detected in recreational water samples on 8/20 occasions (40%).<br>● ESBL-EC were present in all WW samples in ratios of 0.56–0.75%.<br>● ST131 was most prevalent in urine & WW, & ST10 was most prevalent in surface water.<br>● 8-STs & identical ESBL-EC MLVA-types were detected in all compartments.<br>● Clinical ESBL-EC isolates were more likely to be MDR.<br>● ESBL-EC were present in recreational waters.<br>● MDR *E. coli* strains were present in urban aquatic system, even where antibiotic consumption in both humans & animals was restricted.<br>● The three compartments represent highly similar populations of ESBL-EC, with some significant differences.<br>● ESBL-ECs detected in WW could approximate the mix of commensal and pathogenic strains carried by humans in the catchment area, but the clinical isolates could represent more virulent strains.<br>● MDR ESBL-EC AST was more common in clinical samples.	(Jørgensen et al., 2017)
3 samples from each site: hospital effluent, MWW influent, MWW effluent, upstream and downstream of recipient lake (Sweden).	Coliforms and other Gram-negative bacteria	● Bacteria were isolated in Chromocult coliform agar and the prevalence of ARGs was monitored with high-throughput qPCR	● ARB isolates in hospital sewage and municipal sewage were mostly detected together.<br>● CARBA genes *bla*$_{IMP-1}$, *bla*$_{IMP-2}$, and *bla*$_{OXA-23}$ were found in hospital sewage, but not in municipal sewage.<br>● Genes *bla*$_{OXA-48}$, *bla*$_{CTX-M-8}$, and *bla*$_{SFC-1}$ were found in municipal sewage, but not in hospital wastewater.	(Khan et al., 2019)
62 raw and 62 treated MWW were collected on 30 sampling days from Zakynthos, Greece.	*E. coli, Staphylococcus* sp., *Pseudomonas* sp. & *Enterococcus* sp.	● Influent and effluent samples were analyzed with a membrane filtration-based approach followed by culturing.<br>● Kirby–Bauer disk diffusion was used for ARB testing.	● Mostly no difference in ARB profile before and after WWT.<br>● ARB in WWTP reflected the patterns of antibiotic use in the home countries of visitors.<br>● ARB profiles dropped during the season when northern European tourists mostly visit Greece.	(Kolokotsa & Leotsinidis, 2020)
Sequenced ARB *Campylobacter* from humans, livestock, & urban effluents in Spain.	*C. jejuni* & *C. coli*	● Investigated genomic variation associated with MDR ARB in 168 *C. jejuni* & 92 *E. coli* isolated from humans, livestock, and urban effluents.<br>● Isolated *Campylobacter* related to campylobacteriosis cases from hospital sewersheds (n = 152).<br>● Comparison of wastewater *Campylobacter* isolates and clinical isolates.	● Specific ARGs have spread among *Campylobacter* isolates from humans, animals, and the environment.<br>● Phenotypic resistance profiles of individual antibiotics tested were similar between human and sewage isolates, but genotypic comparisons revealed that human isolates generally had a higher prevalence of ARG.<br>● Zoonotic bacteria do not reside in a single host niche; therefore, the source and sink dynamics of resistant strains may be poorly understood.	(Mourkas et al., 2019)
Presented a comprehensive approach to a clonal diversity analysis of 448 ESBL-producing *E. coli* isolated from environmental, human, & food samples in Spain.	ESBL *E. coli*	● Samples were collected from food (n = 580) and the environment (n = 592). Isolates were analyzed from food, mostly fresh meat (n = 179), WWTPs (n = 132), rivers (n = 30), farms (n = 46), and humans (n = 130).<br>● Disk diffusion and microdilution methods were used for phenotypic confirmation of ESBL *E. coli*.<br>● PCR & sequencing were used for the molecular characterization of β-lactamase genes (*bla*$_{CTX-M}$, *bla*$_{SHV}$, *bla*$_{TEM}$, *bla*$_{OXA}$).<br>● The clonal relationship of isolates was determined by multi-locus sequence typing (MLST).	● MDR strains were present in all niches, with a prevalence of >50.0%.<br>● The most prevalent β-lactamase genes were *bla*$_{CTXM-14}$ (26%) & *bla*$_{CTXM-1}$ (21.4%), followed by *bla*$_{SHV-12}$, *bla*$_{CTX-M-15}$, & *bla*$_{TEM-42}$.<br>● MLST isolates were grouped into 26 clonal complexes (CC) & 177 different sequence types (ST).<br>● Despite the high clonal diversity observed, CC10 was the most prevalent CC & the only one detected in all niches.<br>● As ESBL was detected in all sampled niches, the One Health approach could be the best approach to preventing and controlling ESBL.	(Ojer-usoz et al., 2017)

Czech Republic. Isolates from WW effluent, hospital surface swabs, clinical fecal samples, gull cloacal swabs were compared.	Vancomycin-resistant *Enterococci* (VRE)	● WW (n = 37), hospital surface swabs (n = 21), fecal samples from hospitalized patients & staff (n = 59), & gull cloacal swabs (n = 284).<br>● Selectively cultured & Enterococci were confirmed with MALDI-TOF MS.<br>● ARB was confirmed with PCR and genotype diversity was monitored with PFGE & MLST.	● VRE carrying the *vanA* gene were found in 32/37 (86%) WW samples, with a total of 49 isolates, and most of them (44/49) were *E. faecium*.<br>● VRE carrying the *vanA* gene were found in 33/48 (69%) stool samples, with a total of 39 isolates, and most of them (33/39) belonged to *E. faecium*.<br>● 67/80 (84%) *E. faecium* isolates carried virulence genes *hyl* and/or *esp*.<br>● Virulence of *E. faecalis* was encoded by *gelE*, *asa1*, & *cylA* genes.<br>● Clinically relevant sequence types belonging to *E. faecium* were frequently detected in both WW and hospital samples.<br>● The clinically important ARG *vanA* was frequently detected in WW effluent.	(Oravcova et al., 2017)
A total of 108 WW samples were collected from HW, CW, and UW.	*E. coli*	● *E. coli* from HW (n = 2644), CW (n = 2525), and UW (n = 2693) were analyzed for susceptibility to nine antibiotics.	● High levels of resistant *E. coli* were found in hospital and community wastewater, but lower levels in the WWTP.<br>● A higher diversity of *E. coli* was found in community WW than in HWW.	(Paulshus et al., 2019)
At the local scale from a WWTP in Sicily (Italy).	Ampicillin-resistant *Salmonella* spp. *E. coli*-related genes	● *Salmonella* spp. isolates from MWW (n = 64), clinical samples (n = 274), and *E. coli* WW (n = 273) analyzed.	● Ampicillin resistance in *Salmonella* isolates from WW and clinical isolates was comparable. The resistance rate was highly variable in different hosts.	(Pignato et al., 2010)
Determined the epidemiological relatedness between MRSA isolated from sewage and human infection in Tehran, Iran.	Methicillin-resistant *Staphylo coccus aureus*	● Three samples were collected.<br>● Samples were five-fold diluted with phosphate-buffered saline, filtered on a 0.45 µm membrane & cultured in Baird Parker agar.<br>● Specimens (n = 489) were collected from clinical patients.<br>● Susceptibility testing (amikacin, chloramphenicol, ciprofloxacin, clindamycin, erythromycin, gentamicin, kanamycin, linezolid, minocycline, nitrofurantoin, penicillin, quinupristin-dalfopristin, rifampicin, sulfamethoxazole-trimethoprim, tetracycline, and tobramycin) was performed with CLSI guidelines.<br>● Prevalence of ARGs was confirmed with multiplex PCR/PCR *SCCmec* genes & *ccr* gene complexes.	● Of the 1142 isolates, 200 MRSA strains from sewage (n = 100) and the clinic (n = 100) were isolated.<br>● Distinct PhP types, consisting of 16 common types & 13 single types, & 3 different staphylococcal cassette chromosome *mec* (SCCmec) types (III, IVa and IVc) were found amongst the MRSA isolated from the two different sources.<br>● The results of antibiotic susceptibility testing demonstrated increased resistance to penicillin, ciprofloxacin, erythromycin, clindamycin, and tetracycline.<br>● The presence of common PhP types and *SCCmec* type III, as an indicator for hospital strains, among the isolates might indicate an epidemiological link between clinical and sewage MRSA isolates.<br>● Illustrated the presence & persistence of a highly resistant MRSA group in sewage & in clinical samples.	(Rahimi & Bouzari, 2015)
Examined whether WW surveillance could capture clinical cases in communities in the east of England.	*E. coli*	● Treated and untreated WW was taken from 20 WWTPs.<br>● Sequenced 388 *E. coli* isolates (192 ESBL, 196 non-ESBL).<br>● Compared the genomes of WW *E. coli* with isolates from bloodstream infection (n = 437), and livestock farms and retail meat (n = 431).	● All samples were positive in culture for *E. coli*, and all but one was positive for ESBL-producing *E. coli*.<br>● WWT (including UV light) did not eradicate ESBL-*E. coli* in 2/3 cases.<br>● Multilocus sequence type (ST) diversity was similar between plants directly receiving hospital waste versus the remainder (93 vs 95 STs, respectively).<br>● A total of 19/20 wastewater plants contained one or more of the three most common STs associated with bloodstream infection (ST131, ST73, ST95), and 14/20 contained the most common livestock ST (ST10).<br>● In an analysis of 1254 genomes (2 cryptic *E. coli* were excluded), WW isolates were distributed across the phylogeny and intermixed with isolates from humans and livestock.<br>● Ten $bla_{CTX\text{-}M}$ elements were identified in *E. coli* isolated from WW.<br>● Genomic surveillance of *E. coli* in WW could be used to monitor new and circulating lineages and resistance determinants of public-health importance.	(Raven et al., 2019)
24 samples collected from 4 stages of WWT	Carbapenemase-producing GNB (*acrB, adeG,*	● WW samples were analyzed with metagenomics.<br>● Clinical data were obtained from previously published studies from clinical samples.	● MDR genes (*acrB, adeG, mexD* & *mexK*) were the most abundant<br>● Clinically important ARGs such as bla_{KPC}- & $bla_{CTX\text{-}M}$, & others clinically not reported locally, $bla_{TEM\text{-}196}$, $bla_{GES\text{-}23}$, $bla_{OXA\text{-}10}$, *mcr*-3, & *mcr*-5 were frequently detected.	(Rodríguez et al., 2021)

were analyzed (Antioquia, Colombia).	*mexD, mexK*, bla_{KPC}, bla_{CTX-M}, $bla_{TEM-196}$, bla_{GES-23}, bla_{OXA-10}, *mcr*-3, *mcr*-5)	● Initially isolated on ChromID CARBA and ChromoID ESBL media, then sub-cultured on MacConkey agar to monitor potential genes with multiplexed PCR.	● Clinically relevant families *Moraxellaceae*, *Aeromonadaceae,* & *Enterobacteriaceae* were detected.<br>● Clinically relevant genes detected from phenotypic bacterial isolates were also obtained in metagenomic analysis.	
Influent from three WWTPs in Tehran, Iran.	High-level gentamicin-resistant *Enterococcus faecium* (HLGR-EF)	● Disk-diffusion and microbroth dilution for antibiotic susceptibility testing (10 antibiotics).<br>● ARGs were confirmed with PCR.<br>● A total of 106 HLGR-EF (clinical = 48, WW = 58)<br>● 320 enterococcal isolates cultured from urine & wound samples from 6 outpatient clinics during the sampling period.<br>● Clonality was investigated with the PhePlate system (PhP), ribotyping & pulsed-field gel electrophoresis (PFGE).<br>● Representative isolates of each PhP type (n = 42) were further characterized using the ribotyping method.	● PFGE recognized 24 clonal types among these isolates with three pulsotypes shared between the clinical and WW isolates.<br>● 16 ribotypes were identified and 5 types of them were common between clinical & WW isolates.<br>● The combination of PhP typing, ribotyping, and PFGE could be extremely discriminatory when examining HLGR-EF isolates.<br>● WW & clinical isolates were from same city, but overlap was low.<br>● Clinical isolate resistance rates were higher than wastewater isolates. Individual resistances matched national prescribing levels. The low PFGE type overlapped between groups.	(Saifi et al., 2009)
Three MWWTPs, two HWWTPs, and urface water from different parts of Tehran, Iran.	Vancomycin-resistant *E. faecium* (VREF)	● VREF isolates were obtained from patients (n = 49), surface water (n = 28), and urban and hospital sewage (n = 66).<br>● Pulsed-field gel electrophoresis (PFGE) was used to reveal the *E. faecium* clone structure.<br>● The susceptibility to vancomycin was determined using the disc diffusion method and MIC was determined with Etest.<br>● Clinical samples (urine, wound specimens, blood, body fluids, respiratory tract specimens, and abscesses) were collected from hospitalized patients in three major hospitals.<br>● Sewage samples were cultured on vancomycin-supplemented M-Enterococcus agar plates.	● The genetic diversity determined using pulsed-field gel electrophoresis (PFGE) revealed a single *E. faecium* clone, designated 44, which was common to the samples obtained from clinical specimens and hospital and municipal sewage.	(Talebi et al., 2008)
WW effluent samples from Helsinki, Finland.	Carbapenemase-producing GNB	● A total of 7 WW influent samples were analyzed.<br>● ChromID ESBL & ChromID KPC was used for isolation and culture of bacteria.<br>● Multiplexed PCR was used to confirm the prevalence of selected Carbapenemase types.<br>● bla_{CARBA} positive isolates were identified with MALDI-TOF MS.	● Among the carba-GNB isolates, 86.4% were bla_{GES} (51 out of 59), 10.2% were bla_{KPC} (6 out of 59), and 3.4% were bla_{VIM} (2 out of 59).<br>● The bla_{GES} isolates were not detected in clinical isolates.<br>● The most common carba gene, bla_{GES}, was carried by ten different bacterial species, including *Aeromonas* spp., *Enterobacter* spp., and *Kluyvera* spp.; the bla_{KPC} gene was carried by *E. coli*, *K. pneumoniae*, and *Kluyvera cryocescens*; and the bla_{VIM} gene was carried by *Aeromonas hydrophila/caviae* and *Citrobacter amalonaticus*.<br>● WWS can be an additional tool for monitoring ARB at the population level.	(Tiwari et al., 2022)
One-health perspective was considered, and sampling was conducted over 2 years in Canada.	*Enterococcus* spp.	● Isolates from humans & cattle, along with abattoirs, manured fields, natural streams, & composite WW from urban & cattle feedlot sources were collected.<br>● A total of 8430 isolates were collected<br>● Selective culture, disk diffusion, and WGS of a subset of isolates.<br>● Comparison of WW isolates, clinical isolates, and isolates from animal farms.<br>● Clinical isolates mostly from non-sterile sites, but also from blood, other tissues, and rectal swabs (subset for WGS = 299).	● *E. faecium* & *E. faecalis* were the main species in WW (90%) & clinical samples (99%).<br>● *E. hirae* predominated in cattle (92%) & feedlot catch-basins (60%).<br>● WGS of *E. faecalis* (n = 366) & *E. faecium* (n = 342) isolates revealed source clustering of isolates, indicative of distinct adaptation to their respective environments.<br>● Phenotypic resistance to tetracyclines and macrolides encoded by *tet(M)* & *erm(B)*, respectively, was prevalent in *Enterococcus* spp., regardless of the source.<br>● *E. faecium* from cattle that were resistant to β-lactams & quinolones were observed among 3% & 8% of isolates, respectively, compared to 76% and 70% of human clinical isolates.<br>● Clinical vancomycin-resistant *E. faecium* exhibited high rates of multi-drug resistance, with resistance to all β-lactams, macrolides, & quinolones tested.	(Zaheer et al., 2020)

			● Differences in the ARB profiles among isolates reflected antibiotic use practices in each sector of the One Health continuum.	
Activated sludge from 5 WWTPs (grab samples), Austria.	ESBL *E. coli*	● 30 sludge samples were analyzed and 50 ESBL *E. coli* were isolated<br>● ESBL *E. coli* were isolated from 50 patients<br>● ESBL-positive *E. coli* were confirmed by CLSI confirmatory tests<br>● Susceptibility to 11 antibiotics (amoxicillin/clavulanic acid, piperacillin/tazobactam, imipenem, meropenem, gentamicin, tobramycin, amikacin, trimethoprim/sulfamethoxazole, nitrofurantoin, ofloxacin and ciprofloxacin) was tested with Vitek 2.<br>● Susceptibilities to nalidixic acid, tetracycline, and chloramphenicol were tested with disc diffusion following CLSI criteria.<br>● Prevalence of five different β-lactamase gene families (bla_{TEM}, bla_{SHV}, $bla_{CTX\text{-}M}$, bla_{VEB} & bla_{GES}) was tested with PCR.	● The dominant ESBL gene family in clinical and WW isolates was $bla_{CTX\text{-}M}$, one of the most prevalent ESBL gene families in human infection.<br>● The detection rate of the $bla_{TEM\text{-}1}$ gene was similar in clinical and WW isolates.<br>● Higher resistance rates against clinical antibiotics were seen in UTI isolates but not for non-clinical antibiotics.<br>● Resistance to the antibiotic groups ampicillin/clavulanic acid, tobramycin, amikacin, trimethoprim/sulfamethoxazole, ciprofloxacin, ofloxacin, and nalidixic acid were significantly higher in clinical isolates than in WW-based isolates.	(Zarfel et al., 2013)
Cohort study conducted in Vietnam, sampled the resistome of human feces, animal stools, human food, and environmental samples.	$bla_{CTX\text{-}M\text{-}1}$, $bla_{CTX\text{-}M\text{-}2}$, $bla_{CTX\text{-}M\text{-}9}$, bla_{NDM}, bla_{VIM}, bla_{KPC}, $bla_{OXA\text{-}48}$, *mcr*-1, *mcr*-2, mcr-3, *mrc*-4 & *mcr*-5.	● A total of 304 samples were analyzed.<br>● Selected 80 households (359 people) and followed their antibiotic consumption, as well as antibiotic contents in food & water for four months and analyzed ARGs in their feces.<br>● Extracted DNA & monitored resistance genes related to mobile colistin resistance (*mcr*), ESBL, CARB, & quinolone with qPCR.	● Nearly 40% of samples contained ESBL genes (the most frequent were *CTX-M-9* (23.7%) and *CTX-M-1* (18.8%)).<br>● Quinolone resistance genes (*qnrS*) were detected in all human and 91% of animal stool samples.<br>● *Mcr-1* was present in 88% of human feces & 93% of animal feces samples.<br>● *Mcr-3* was present in 55% of human feces & 51% of animal feces samples.<br>● CARBA genes were more common in water (15%) and cooked food (13%) than in human and animal feces (<4%).<br>● It was not possible to establish a relationship between recent antibiotic consumption and ARGs in human stool samples.<br>● PCA analysis demonstrated a clear separation between ARG profiles of human and animal stools versus cooked food and water samples.	(Bich et al., 2019)
Three WWTPs in South Africa.	*Mycobacterium tuberculosis*	● Conventional PCR & ddPCR were used.<br>● Tuberculosis-related genes were targeted and compared with the extent of community drug use.	● dPCR was more sensitive than qPCR.<br>● The concentration of ARGs was associated with the extent of drug use in communities.	(Mtetwa et al., 2021)
Urban sewage was monitored for the presence of pathogens and ARG in Kibera, Nairobi, Kenya	ARG Metagenome	● Metagenomic analysis of urban sewage to monitor bacterial & associated ARG, viral and parasitic pathogens. Data were examined in conjunction with data from ongoing clinical infectious disease surveillance.	● WW metagenomics can be a potential public health surveillance tool with high sensitivity and can be a valuable supplementary tool for clinical and syndromic surveillance of large urban populations.<br>● Large variation in read abundances related to bacteria, viruses, and parasites of medical importance, as well as bacterial-associated antibiotic resistance genes, was reported over time.<br>● WW metagenomics may provide an opportunity for conducting real-time global genomics-based surveillance in settings with limited access to health care.	(R. Hendriksen et al., 2019)
Influent and effluent samples, 8 from each, with an 18-month duration. (USA)	ARG Metagenome	● Metagenomics analysis was compared with local clinical resistant isolates<br>● Metagenomics analysis was compared and bench-marked to various dimensions of the resistome, as they related to independent quantitative measures of target ARGs, factors associated with antibiotic use in the community and ARG selection (e.g., antibiotics), mobility (e.g., associations with MGEs), and local clinical resistance information.	● Metagenomics analysis was found to yield rich information comparable to clinical isolates.<br>● The relative abundance of total ARGs dropped ~50% from influent to effluent.<br>● ~90% of the ARGs found in the effluent were also detected in the influent.<br>● The effluent ARG-pathogen taxonomic linkage patterns were more similar to patterns in regional clinical surveillance data than the patterns identified in the influent.	(Majeed et al., 2021)

		• Examined the core resistome, discriminatory resistomes, specific ARGs of clinical concern, and resistome risk scores and compared these with independent qPCR measurements of target ARGs, antibiotic measurements, and local clinical resistance data.		
**Hospital level (Single building level)**				
24-h composite samples from a geriatric hospital in France.	ESBL-producing *Enterobacteriaceae*	• ESBL-producing *Enterobacteriaceae* were isolated with chromID ESBL.<br>• Clinical samples were analyzed either with clinical specimens or rectal swabs.	• A total of six distinct environmental ESBL *E. coli* clusters were identified, two of them related to patient strains.	(Drieux et al., 2016)
20 composite HWW samples were collected for 2 years in a hospital in Gothenburg, Sweden.	Carbapenemase-producing *Enterobacterales*	• A total of 20 WW composite samples were compared with about 14,000 urine samples, 11,000 blood samples, and 2000 fecal rectal clinical samples for the same ARB.<br>• ARB in WW was monitored with both cell isolation and qPCR, and WGS of some isolates was carried out.	• The carbapenemase genes bla_{NDM}, $bla_{OXA\text{-}48\text{-}like}$, and bla_{KPC} in sewage were concordant with clinical cases, but the genes bla_{VIM} and bla_{IMP} were detected with qPCR of WWW but not in WW isolates.<br>• Three clinical patients in a hospital caused a peak in related genes in sewage for the $bla_{OXA\text{-}48\text{-}like}$ gene.	(Flach et al., 2021)
Hospital clinical isolates and HWW, Taiwan.	*E. coli*	• ARB of a total of 1020 clinical & 435 sewage isolates was tested with the Kirby–Bauerdisk diffusion test for 8 antibiotics (ampicillin, ampicillin⁄ sulbactam, cefazolin, cefuroxime, imipenem, lomefloxacin, gentamicin, & amikacin).	• Sewage isolates had higher ARB rates than the clinical isolates from the same hospital.	(Yang et al., 2009)
A total of 30 hospital sewage samples were collected from Shantou University Hospital, China.	Evaluated association between antibiotic use in hospital, AR information from clinical isolates, & ARB/ARG profiles in the hospital sewage.	• ARB were isolated with selective media.<br>• ARGs were monitored with metagenomics.	• ARGs and ARB in clinical isolates had a strong relationship with clinical isolates.<br>• Hospital antibiotic use influenced the abundance and types of ARB and ARG.<br>• ARGs released from sewage can increase environmental and public health risks.<br>• The culture-based method captured 16.2% of ARB species, but metagenomics captured 1573 bacterial species and 885 types of ARG in sewage.<br>• Metagenomics analysis revealed that ARGs for aminoglycosides were the most common, followed by sulphonamide, tetracycline, phenicol, macrolides, and quinolones, together representing 82.6% all ARGs.<br>• A total of 519 pairs of ARGs were significantly correlated with ARB species ($r > 0.8$).	(Cai et al., 2021)
HWW (n = 8) was collected from 8 different departments of a hospital and one sample from MWW (Western General Hospital, Edinburgh, Scotland).	ARG Metagenome	• Metagenomic analysis<br>• Correlation between ARG abundances in HWW was tested with hospital antibiotic use, hospital clinical isolates, and isolates from MWW.	• HWW had a higher ARG abundance than MWW.<br>• ARG abundances in HWW did not reflect resistance patterns in clinical isolates, except for the families *Enterococcaceae* & *Staphylococcaceae*.<br>• Antibiotic use was not associated with ARG abundance in HWW, except for carbapenem & vancomycin resistance ARG.	(Perry et al., 2021)

AMR = antimicrobial resistance, ARG = antibiotic resistance genes, HWW = hospital wastewater, MWW = municipal wastewater, MDR = multidrug-resistant, PFGE = pulsed-field gel electrophoresis, MLST = multi-locus sequence typing, MRSA = Methicillin-resistant *Staphylococcus aureus*, WW = wastewater, ESBL = extended spectrum beta-lactamase

References

Abduljabbar, A., & Aljanaby, J. (2018). Antibiotics Susceptibility Pattern and Virulence-associated Genes in Clinical and Environment Strains of Pseudomonas aeruginosa in Iraq. *Asian J. Sci. Res.*, *11*, 401–408. https://doi.org/10.3923/ajsr.2018.401.408

Adator, E. H., Narvaez-bravo, C., Zaheer, R., Cook, S. R., Tymensen, L., Hannon, S. J., Booker, C. W., Church, D., Read, R. R., & Mcallister, T. A. (2020). A One Health Comparative Assessment of Antimicrobial Resistance in Generic and Escherichia coli from Beef Production , Sewage and Clinical Settings. *Microorganisms*, *8*(885), 1–22.

Auguet, O. T., Niehus, R., Gweon, H. S., Berkley, J. A., Waichungo, J., Njim, T., Edgeworth, J. D., Batra, R., Chau, K., Swann, J., Walker, S. A., Peto, T. E. A., Crook, D. W., Lamble, S., Turner, P., Cooper, B. S., & Stoesser, N. (2021). Population-level faecal metagenomic profiling as a tool to predict antimicrobial resistance in Enterobacterales isolates causing invasive infections: An exploratory study across Cambodia, Kenya, and the UK. *EClinicalMedicine*, *36*, 100910. https://doi.org/10.1016/j.eclinm.2021.100910

Berendonk, T. U., Manaia, C. M., Merlin, C., Kassinos, D. F., Cytryn, E., Walsh, F., Bürgmann, H., Huovinen, P., Stefani, S., Schwartz, T., Kisand, V., Baquero, F., & Martinez, J. L. (2015). Tackling antibiotic resistance: the environmental framework. *Nature Reviews Microbiology*, *13*, 310–317. https://doi.org/10.1038/nrmicro3439

Bich, V. T. N., Thanh, L. V., Thai, P. D., Phuong, T. T. Van, Oomen, M., Driessen, C., Beuken, E., Hoang, T. H., Doorn, H. R. Van, Penders, J., & Wertheim, H. F. L. (2019). An exploration of the gut and environmental resistome in a community in northern Vietnam in relation to antibiotic use. *Antimicrobial Resistance and Infection Control*, *8*(194). https://doi.org/https://doi.org/10.1186/s13756-019-0645-9

Blaak, H., Kemper, M. A., de Man, H., van Leuken, J. P. G., Schijven, J. F., van Passel, M. W. J., Schmitt, H., & de Roda Husman, A. M. (2021). Nationwide surveillance reveals frequent detection of carbapenemase-producing Enterobacterales in Dutch municipal wastewater. *Science of the Total Environment*, *776*. https://doi.org/10.1016/j.scitotenv.2021.145925

Cai, L., Sun, J., Yao, F., Yuan, Y., Zeng, M., Zhang, Q., Xie, Q., Wang, S., Wang, Z., & Jiao, X. (2021). Antimicrobial resistance bacteria and genes detected in hospital sewage provide valuable information in predicting clinical antimicrobial resistance. *The Science of the Total Environment*, *795*, 148815. https://doi.org/10.1016/j.scitotenv.2021.148815

Diemert, S., & Yan, T. (2019). Clinically Unreported Salmonellosis Outbreak Detected via Comparative Genomic Analysis of Municipal Wastewater Salmonella Isolates. *Applied and Environmental Microbiology*, *85*(10), 1–11. https://doi.org/10.1128/AEM.00139-19

Drieux, L., Haenn, S., Moulin, L., & Jarlier, V. (2016). Quantitative evaluation of extended-spectrum β -lactamase-producing Escherichia coli strains in the wastewater of a French teaching hospital and relation to patient strain. *Antimicrobial Resistance & Infection Control*, *5*(9), 1–5. https://doi.org/10.1186/s13756-016-0108-5

Flach, C.-F., Hutinel, M., Razavi, M., Åhrén, C., & Larsson, D. G. J. (2021). Monitoring of hospital sewage shows both promise and limitations as an early-warning system for carbapenemase-producing Enterobacterales in a low-prevalence setting. *Water Research*, *200*, 117261. https://doi.org/10.1016/j.watres.2021.117261

Forslund, K., Sunagawa, S., Kultima, J. R., Mende, D. R., Arumugam, M., Typas, A., & Bork, P. (2013). Country-specific antibiotic use practices impact the human gut resistome. *Genome*

Research, *23*, 1163–1169. https://doi.org/10.1101/gr.155465.113.23

Golle, A., Janezic, S., & Rupnik, M. (2017). Low overlap between carbapenem resistant Pseudomonas aeruginosa genotypes isolated from hospitalized patients and wastewater treatment plants. *PLoS ONE*, *12*(10), e0186736.

Gouliouris, T., Raven, K. E., Moradigaravand, D., Ludden, C., Coll, F., Blane, B., Naydenova, P., Horner, C., Brown, N. M., Corander, J., Limmathurotsakul, D., Parkhill, J., & Peacock, S. J. (2019). Detection of vancomycin-resistant Enterococcus faecium hospital-adapted lineages in municipal wastewater treatment plants indicates widespread distribution and release into the environment. *Genome Research*, *29*, 626–634. https://doi.org/10.1101/gr.232629.117.Freely

Haghi, F., Shirmohammadlou, N., Bagheri, R., Jamali, S., & Zeighami, H. (2019). High Frequency of Vancomycin-Resistant Enterococci in Sewage and Fecal Samples of Healthy Carriers Abstract : *The Open Biotechnology Journal*, *13*, 1–5. https://doi.org/10.2174/1874070701913010001

Hendriksen, R., Lukjancenko, O., Id, P. M., Hjelms, M. H., Verani, J. R., Ng, E., Bigogo, G., Kiplangat, S., Neatherlin, J. C., Clayton, O., Hald, T., Bergmark, L., Ro, T., Pamp, J., Fields, B., Montgomery, J. M., & Karlsmose, S. (2019). Pathogen surveillance in the informal settlement, Kibera, Kenya, using a metagenomics approach. *PLoS One*, *14*(10), e0222531.

Hendriksen, R. S., Munk, P., Njage, P., van Bunnik, B., McNally, L., Lukjancenko, O., Röder, T., Nieuwenhuijse, D., Pedersen, S. K., Kjeldgaard, J., Kaas, R. S., Clausen, P. T. L. C., Vogt, J. K., Leekitcharoenphon, P., van de Schans, M. G. M., Zuidema, T., de Roda Husman, A. M., Rasmussen, S., Petersen, B., … Aarestrup, F. M. (2019). Global monitoring of antimicrobial resistance based on metagenomics analyses of urban sewage. *Nature Communications*, *10*(1), 1124. https://doi.org/10.1038/s41467-019-08853-3

Huijbers, P. M. C., Larsson, D. G. J., & Flach, C. F. (2020). Surveillance of antibiotic resistant Escherichia coli in human populations through urban wastewater in ten European countries. *Environmental Pollution*, *261*. https://doi.org/10.1016/j.envpol.2020.114200

Hutinel, M., Huijbers, P. M. C., Fick, J., Åhrén, C., Larsson, D. G. J., & Flach, C.-F. (2019). Population-level surveillance of antibiotic resistance in Escherichia coli through sewage analysis. *Eurosurveillance*, *24*(37), 1–11. https://doi.org/10.2807/1560-7917.ES.2019.24.37.1800497

Jakobsen, L., Sandvang, D., Hansen, L. H., Bagger-skjøt, L., Westh, H., Jørgensen, C., Hansen, D. S., Pedersen, B. M., Monnet, D. L., Frimodt-møller, N., Sørensen, S. J., & Hammerum, A. M. (2008). Characterisation, dissemination and persistence of gentamicin resistant Escherichia coli from a Danish university hospital to the waste water environment. *Environment International*, *34*(1), 108–115. https://doi.org/10.1016/j.envint.2007.07.011

Jørgensen, S., Søraas, A., Arnesen, L., Leegaard, T., Sundsfjord, A., & Jenum, P. (2017). A comparison of extended spectrum β -lactamase producing Escherichia coli from clinical , recreational water and wastewater samples associated in time and location. *PLoS ONE*, *12*(10), e0186576. https://doi.org/https://doi.org/10.1371/journal.pone.0186576 October

Karkman, A., Berglund, F., Flach, C.-F., Kristiansson, E., & Larsson, D. G. J. (2020). Predicting clinical resistance prevalence using sewage metagenomic data. *Communications Biology*, *3*(1), 711. https://doi.org/10.1038/s42003-020-01439-6

Khan, F. A., Söderquist, B., & Jass, J. (2019). Prevalence and diversity of antibiotic resistance genes in Swedish aquatic environments impacted by household and hospital wastewater. *Frontiers in Microbiology*, *10*(APR), 1–12. https://doi.org/10.3389/fmicb.2019.00688

Kolokotsa, A., & Leotsinidis, M. (2020). Effects of tourist flows on antibiotic resistance in wastewater of a Greek island. *Journal of Applied Microbiology*, *130*, 516–527. https://doi.org/10.1111/jam.14808

Majeed, H. J., Riquelme, M. V., Davis, B. C., Gupta, S., Angeles, L., Aga, D. S., Garner, E., Pruden, A., & Vikesland, P. J. (2021). Evaluation of Metagenomic-Enabled Antibiotic Resistance Surveillance at a Conventional Wastewater Treatment Plant. *Frontiers in Microbiology*, *12*(May), 1–19. https://doi.org/10.3389/fmicb.2021.657954

Meir-Gruber, L., Manor, Y., Gefen-Halevi, S., Hindiyeh, M., Mileguir, F., Azar, R., Smollan, G., Belausov, N., Rahav, G., Shamiss, A., Mendelson, E., & Keller, N. (2016). Population Screening Using Sewage Reveals Pan-Resistant Bacteria in Hospital and Community Samples. *PLoS One*, *11*(10), e0164873. https://doi.org/10.1371/journal.pone.0164873

Moradigaravand, D., Gouliouris, T., Ludden, C., Reuter, S., Jamrozy, D., Blane, B., Naydenova, P., Judge, K., Aliyu, S. H., Hadjirin, N. F., & Holmes, M. A. (2018). Genomic survey of Clostridium difficile reservoirs in the East of England implicates environmental contamination of wastewater treatment plants by clinical lineages. *Microbial Genomics*, *4*. https://doi.org/10.1099/mgen.0.000162

Mourkas, E., Florez-cuadrado, D., Pascoe, B., Calland, J. K., Bayliss, S. C., Mageiros, L., Méric, G., Hitchings, M. D., Quesada, A., Porrero, C., Ugarte-ruiz, M., Gutiérrez-fernández, J., Domínguez, L., & Sheppard, S. K. (2019). Gene pool transmission of multidrug resistance among Campylobacter from livestock, sewage and human disease. *Environmental Microbiology*, *21*(12), 4597–4613.

Mtetwa, H. N., Amoah, I. D., Kumari, S., Bux, F., & Reddy, P. (2021). Wastewater-Based Surveillance of Antibiotic Resistance Genes Associated with Tuberculosis Treatment Regimen in KwaZulu Natal, South Africa. *Antibiotics*, *10*(11), 1362. https://doi.org/10.3390/antibiotics10111362

Ojer-usoz, E., Gonz, D., & Vitas, A. I. (2017). Clonal Diversity of ESBL-Producing Escherichia coli Isolated from Environmental, Human and Food Samples. *Int. J. Environ. Res. Public Health*, *14*(676). https://doi.org/10.3390/ijerph14070676

Oravcova, V., Mihalcin, M., Zakova, J., Pospisilova, L., Masarikova, M., & Literak, I. (2017). Vancomycin-resistant enterococci with vanA gene in treated municipal wastewater and their association with human hospital strains. *Science of the Total Environment*, *609*, 633–643. https://doi.org/10.1016/j.scitotenv.2017.07.121

Pärnänen, K., Narciso-da-Rocha, C., Kneis, D., Berendonk, T. U., Cacace, D., Do, T. T., Elpers, C., Fatta-Kassinos, D., Henriques, I., Jaeger, T., Karkman, A., Martinez, J. L., Michael, S. G., Michael-Kordatou, I., O'Sullivan, K., Rodriguez-Mozaz, S., Schwartz, T., Sheng, H., Sørum, H., … Manaia, C. M. (2019). Antibiotic resistance in European wastewater treatment plants mirrors the pattern of clinical antibiotic resistance prevalence. *Science Advances*, *5*(3), eaau9124. https://doi.org/10.1126/sciadv.aau9124

Paulshus, E., Kuhn, I., Mollby, R., Colque, P., Sullivan, K. O., Midtvedt, T., Lingaas, E., Holmstad, R., & Sorum, H. (2019). Diversity and antibiotic resistance among Escherichia coli populations in hospital and community wastewater compared to wastewater at the receiving urban treatment plant. *Water Research*, *161*, 232–241. https://doi.org/https://doi.org/10.1016/j.watres.2019.05.102

Perry, M. R., Lepper, H. C., McNally, L., Wee, B. A., Munk, P., Warr, A., Moore, B., Kalima, P., Philip, C., de Roda Husman, A. M., Aarestrup, F. M., Woolhouse, M. E. J., & van Bunnik, B. A. D. (2021). Secrets of the Hospital Underbelly: Patterns of Abundance of Antimicrobial

Resistance Genes in Hospital Wastewater Vary by Specific Antimicrobial and Bacterial Family. *Frontiers in Microbiology*, *12*(September). https://doi.org/10.3389/fmicb.2021.703560

Petersen, T. N., Rasmussen, S., Hasman, H., Carøe, C., Bælum, J., Schultz, A. C., Bergmark, L., Svendsen, C. A., Lund, O., Sicheritz-pontén, T., & Aarestrup, F. M. (2015). Meta-genomic analysis of toilet waste from long distance flights ; a step towards global surveillance of infectious diseases and antimicrobial resistance. *Scientific Reports*, *11444*(April), 1–9. https://doi.org/Doi:10.1038/srep11444

Pignato, S., Coniglio, M. A., & Faro, G. (2010). Molecular Epidemiology of Ampicillin Resistance in Salmonella spp . and Escherichia coli from Wastewater. *Foodborne Pathogens And Disease*, *7*(8), 945–951. https://doi.org/10.1089=fpd.2009.0504

Rahimi, F., & Bouzari, M. (2015). Biochemical Fingerprinting of Methicillin-Resistant Staphylococcus aureus Isolated From Sewage and Hospital in Iran. *Jundishapur J Microbiol.*, *8*(7), e19760. https://doi.org/10.5812/jjm.19760v2

Raven, K. E., Ludden, C., Gouliouris, T., Blane, B., Naydenova, P., Brown, N. M., Parkhill, J., & Peacock, S. J. (2019). Genomic surveillance of Escherichia coli in municipal wastewater treatment plants as an indicator of clinically relevant pathogens and their resistance genes. *Microbial Genomics*, *5*. https://doi.org/10.1099/mgen.0.000267

Reinthaler, F. F., Galler, H., Feierl, G., Haas, D., Leitner, E., Mascher, F., Melkes, A., & Posch, J. (2013). Resistance patterns of Escherichia coli isolated from sewage sludge in comparison with those isolated from human patients in 2000 and 2009. *Journal of Water and Health*, *11*(1), 13–20. https://doi.org/10.2166/wh.2012.207

Riquelme, M. V, Garner, E., Gupta, S., Metch, J., Zhu, N., Blair, M. F., Arango-Argoty, G., Maile-Moskowitz, A., Li, A., Flach, C.-F., Aga, D. S., Nambi, I., Larsson, D. G. J., Bürgmann, H., Zhang, T., Pruden, A., & Vikesland, P. J. (2021). Wastewater Based Epidemiology Enabled Surveillance of Antibiotic Resistance. *MedRxiv*, 2021.06.01.21258164. https://doi.org/10.1101/2021.06.01.21258164

Rodríguez, E. A., Ramirez, D., Balcázar, J. L., & Jiménez, J. N. (2021). Metagenomic analysis of urban wastewater resistome and mobilome: A support for antimicrobial resistance surveillance in an endemic country. *Environmental Pollution*, *276*(116736), 116736. https://doi.org/10.1016/j.envpol.2021.116736

Saifi, M., Pourshafie, M., Dallal, M. S., & Katouli, M. (2009). Clonal groups of high-level gentamicin-resistant Enterococcus faecium isolated from municipal wastewater and clinical samples in Tehran, Iran. *Letters in Applied Microbiology*, *49*, 160–165. https://doi.org/10.1111/j.1472-765X.2009.02559.x

Su, J., An, X., Li, B., Chen, Q., Gillings, M. R., Chen, H., & Zhang, T. (2017). Metagenomics of urban sewage identifies an extensively shared antibiotic resistome in China. *Microbiome*, *5*(84), 1–15. https://doi.org/10.1186/s40168-017-0298-y

Talebi, M., Pourshafie, M. R., Katouli, M., & Mo, R. (2008). Molecular Structure and Transferability of Tn 1546 -Like Elements in Enterococcus faecium Isolates from Clinical , Sewage , and Surface Water Samples in Iran. *Applied and Environmental Microbiology*, *74*(5), 1350–1356. https://doi.org/10.1128/AEM.02254-07

Tiwari, A., Paakkanen, J., Österblad, M., Kirveskari, J., Hendriksen, R. S., & Heikinheimo, A. (2022). Wastewater Surveillance Detected Carbapenemase Enzymes in Clinically Relevant Gram-Negative Bacteria in Helsinki, Finland; 2011–2012. *Frontiers in Microbiology*, *13*, 1–14. https://doi.org/10.3389/fmicb.2022.887888

Urase, T., Okazaki, M., & Tsutsui, H. (2020). Prevalence of ESBL-producing Escherichia coli and carbapenem-resistant Enterobacteriaceae in treated wastewater: a comparison with nosocomial infection surveillance. *Journal of Water and Health*, *18*(6), 899–910. https://doi.org/10.2166/wh.2020.014

Yang, C. M., Lin, M. F., Liao, P. C., Yeh, H. W., Chang, B. V., Tang, T. K., Cheng, C., Sung, C. H., & Liou, M. L. (2009). Comparison of antimicrobial resistance patterns between clinical and sewage isolates in a regional hospital in Taiwan. *Letters in Applied Microbiology*, *48*(5), 560–565. https://doi.org/10.1111/j.1472-765X.2009.02572.x

Zaheer, R., Cook, S. R., Barbieri, R., Goji, N., Cameron, A., Petkau, A., Polo, R. O., Tymensen, L., Stamm, C., Song, J., Hannon, S., Jones, T., Church, D., Booker, C. W., Amoako, K., Van Domselaar, G., Read, R. R., & McAllister, T. A. (2020). Surveillance of Enterococcus spp. reveals distinct species and antimicrobial resistance diversity across a One-Health continuum. *Scientific Reports*, *10*(1), 3937. https://doi.org/10.1038/s41598-020-61002-5

Zarfel, G., Galler, H., Feierl, G., Haas, D., Kittinger, C., Leitner, E., Grisold, A. J., Mascher, F., Posch, J., Pertschy, B., Marth, E., & Reinthaler, F. (2013). Comparison of extended-spectrum-beta-lactamase (ESBL) carrying Escherichia coli from sewage sludge and human urinary tract infection. *Environmental Pollution*, *173*, 192–199.
